# Infant Botulism

**DOI:** 10.21980/J8X35W

**Published:** 2022-04-15

**Authors:** Victoria Morris, Robert Wians, Jessica Wilson, Gowri Stevens

**Affiliations:** * McGovern Medical School at the University of Texas Health Science Center, Department of Emergency Medicine, Houston, TX

## Abstract

**Audience:**

Emergency medicine and pediatric residents, and pediatric emergency medicine (PEM) fellows.

**Introduction:**

Botulism is a rare but serious cause of infant hypotonia, vomiting, and respiratory failure. The differential diagnosis and management of a hypotonic infant with progressive weakness leading to respiratory failure is a rare presentation with high morbidity and mortality.^1^ Infants with botulism generally present with vague complaints that progressively worsen over time.^2^ Recognition of descending paralysis in an infant as well as signs of respiratory failure are key to preventing an adverse outcome. A key component of botulism treatment is recognizing the need to mobilize local resources to obtain BabyBIG^®^ (botulism immune globulin). This process can and should begin in the emergency department.

**Educational Objectives:**

After this simulation learners should be able to: 1) develop a differential diagnosis for the hypotonic infant, 2) recognize signs and symptoms of infant botulism, 3) recognize respiratory failure and secure the airway with appropriate rapid sequence intubation (RSI) medications, 4) initiate definitive treatment of infant botulism by mobilizing resources to obtain antitoxin, 5) continue supportive management and admit the patient to the pediatric intensive care unit (PICU), 6) understand the pathophysiology and epidemiology of infant botulism, 7) develop communication and leadership skills when evaluating and managing critically ill infants.

**Educational Methods:**

This simulation case was performed using a high-fidelity Laerdal SimBaby with intubating capabilities and real-time vital sign monitoring. Additionally, this case can be performed with low fidelity manikins with supplemental scripting and visual stimuli. With minor adjustments, this case could be modified into an oral boards case.

**Research Methods:**

We obtained feedback from a convenience sample of random participants after the simulation case and debrief were completed. The sample of emergency medicine residents (N=21) and PEM fellow (N=1) completed 5 questions on a 5-point Likert scale.

**Results:**

The emergency medicine residents and PEM fellow had mostly favorable feedback regarding the simulation and debriefing. Most strongly agreed or agreed that this would improve their performance in an actual clinical setting.

**Discussion:**

Infant botulism is a rare condition, presenting as vague non-specific complaints that worsen over time. It is important to differentiate infant botulism from other causes of weakness, hypotonia, and respiratory failure. This case presents learners with a high acuity, rare case of infant botulism and allows them to work through a complex pediatric patient encounter in a psychologically safe space. The presence of a standardized patient to play the patient’s parent is key to assess learners’ nontechnical communication skills and to increase fidelity during the simulation.

**Topics:**

Infant botulism, pediatric emergency medicine, respiratory failure, hypotonia, toxicology.

## USER GUIDE

**Table:** 

List of Resources:	
Abstract	48
User Guide	50
Case 1: Instructor Materials	52
Case 1: Standardized Patient Briefing Materials	64
Case 1: Operator Materials	67
Case 1: Debriefing and Evaluation Pearls	69
Case 1: Simulation Assessment	73

**Learner Audience:**

Medical students, interns, junior residents, senior residents, PEM fellows

**Time Required for Implementation:**

**Instructor Preparation:** 30 minutes

**Time for case:** 20 minutes

**Time for debriefing:** 20 minutes

**Recommended Number of Learners per Instructor:**

3–4

**Topics:**

Infant botulism, pediatric emergency medicine, respiratory failure, hypotonia, toxicology.

**Objectives:**

At the conclusion of this simulation, learners will be able to:

1. Review the differential diagnosis for the hypotonic infant
2. Recognize signs and symptoms of infant botulism
3. Recognize respiratory failure and secure the airway with appropriate RSI medication and equipment
4. Understand importance of mobilizing resources to procure botulism immune globulin
5. Provide appropriate supportive care and disposition
6. Understand pathophysiology and epidemiology of infant botulism
7. Develop communication and leadership skills

### Linked objectives and methods

Infant botulism is a rare syndrome that has significant morbidity and mortality if not recognized and promptly treated.^1^ Cases of infant botulism present as progressively worsening vague symptoms that may include decreased feeding, hypotonia, and vomiting.^2^ It is important for the emergency medicine physician to be able to diagnose and appropriately treat infant botulism. This simulation will challenge the learners to obtain a thorough history and perform a physical exam to develop a differential diagnosis for hypotonia in an infant (Objective 1) and recognize infant botulism through key points in the history and physical (Objective 2). As the learners pursue a workup for this patient, they should be able to manage the patient’s symptoms of hypoxia and worsening respiratory failure, ultimately intubating the patient (Objective 3). Learners should initiate definitive treatment of infant botulism by mobilizing resources to obtain antitoxin (Objective 4). Once the diagnosis of infant botulism is made and the patient’s airway is secure, the learners should be able to continue supportive management and admit the patient to the pediatric intensive care unit (PICU) (Objective 5). Through the course of this simulation and debrief, learners will better understand the pathophysiology and epidemiology of infant botulism (Objective 6) and develop communication and leadership skills when evaluating and managing critically ill infants (Objective 7).

### Recommended pre-reading for instructor

1. Bodamer O. Neuromuscular Junction Disorders in Newborns and Infants. UpToDate. Published 2021. Accessed February 4, 2022. At: http://www.uptodate.com/contents/neuromuscular-junction-disorders-in-newborns-and-infants
2. Walls RM, Hockberger RS, Gausche-Hill M. Neuromuscular Disorders. In: *Rosen’s Emergency Medicine: Concepts and Clinical Practice*. Philadelphia, PA: Elsevier; 2018:1325–1325.
3. Rosow LK, Strober JB. Infant botulism: review and clinical update. *Pediatr Neurol*. 2015;52(5):487–492. doi:10.1016/j.pediatrneurol.2015.01.006

### Results and tips for successful implementation

This case simulation was designed for residents and fellows to diagnose, treat, and appropriately disposition an infant with botulism and respiratory failure in the emergency department. This scenario was designed to be performed using a high-fidelity pediatric manikin, such as the Laerdal SimBaby. The Laerdal SimBaby was set to pupils of 5 mm and eyes half closed to simulate mydriasis and ptosis. The faculty facilitator adjusted vital sign parameters for the Laerdal SimBaby in real time based upon each groups decision making during the session. This case is augmented by using a standardized patient/confederate to play the part of the parent, who will provide the key points in the history that lead to the diagnosis of infant botulism. The parent can increase fidelity by wiping away secretions. This case was conducted with emergency medicine residents and PEM fellows in groups of 3–4. Feedback was obtained from participants through verbal debriefing and formal survey. A convenience sample of 22 participants completed the survey including emergency medicine residents (N=21) and a PEM fellow (N=1). The survey consisted of 5 questions; 3 questions used a 5-point Likert scale and 2 questions asked for open-ended responses (Table 1). The respondents had mostly favorable feedback regarding the simulation and debriefing. Most strongly agreed or agreed that this scenario would improve their performance in an actual clinical setting. Sample responses to the open-ended questions is provided in Table 2.

### Associated Materials

- Attached is a debriefing PowerPoint to solidify post scenario education.

## Supplementary Information





## Figures and Tables

**Table:** 

#1	Complete blood count (CBC)
#2	Basic metabolic panel (BMP)
#3	Finger stick blood glucose
#4	Venous blood gas (VBG)
#5	Magnesium and Phosphorous
#6	Urinalysis (UA)
#7	Lactate
#8	Chest radiograph
#9	Chest radiograph post intubation
#10	Computed Tomography (CT) brain
#11	Cerebral spinal fluid (CSF) cell count
#12	Cerebral spinal fluid glucose
#13	Cerebral spinal fluid protein

**Table:** 

**Vitals:**	Heart Rate (HR) 175	Blood Pressure (BP) 70/50	Respiratory Rate (RR) 28
	Temperature (T) 37°C	Oxygen Saturation (O_2_Sat) 92% on room air	

**Table:** 

*Complete blood count (CBC)*	
White blood count (WBC)	8.2 x 1000/mm^3^
Hemoglobin (Hgb)	10.2 g/dL
Hematocrit (HCT)	30.5%
Platelet (Plt)	125 x 1000/mm^3^
Segmented Neutrophils	40%
Lymphocytes	20%
Monocytes	3%
Eosinophils	1%
Basophils	0%

**Table:** 

*Basic metabolic panel (BMP)*	
Sodium	138 mEq/L
Potassium	3.5 mEq/L
Chloride	106 mEq/L
Bicarbonate (HCO3)	18 mEq/L
Blood Urea Nitrogen (BUN)	4 mg/dL
Creatinine (Cr)	0.2 mg/dL
Glucose	55 mg/dL
Calcium	9.2 mg/dL

**Table:** 

*Finger-stick blood glucose*	55 mg/dL

**Table:** 

*Venous blood gas (VBG)*	
pH	7.25
PaCO2	55 mmHg
PaO2	60 mmHg
Bicarbonate (HCO3)	19 mEq/L

**Table:** 

*Magnesium*	2 mEq/L

**Table:** 

*Phosphorous*	5 mg/dL

**Table:** 

*Lactic Acid*	1 mmol/L

**Table:** 

*Urinalysis (UA)*	
Color	yellow
Appearance	clear
Specific gravity	1.2
pH	6.5
Glucose	negative
Bilirubin	negative
Ketones	trace
Protein	0
Leukocyte esterase	negative
Nitrites	negative
White blood cells (WBC)	0 WBCs/high powered field (HPF)
Red blood cells (RBC)	0 RBCs/HPF
Squamous epithelial cells	0–5 cells/HPF

**Figure:**
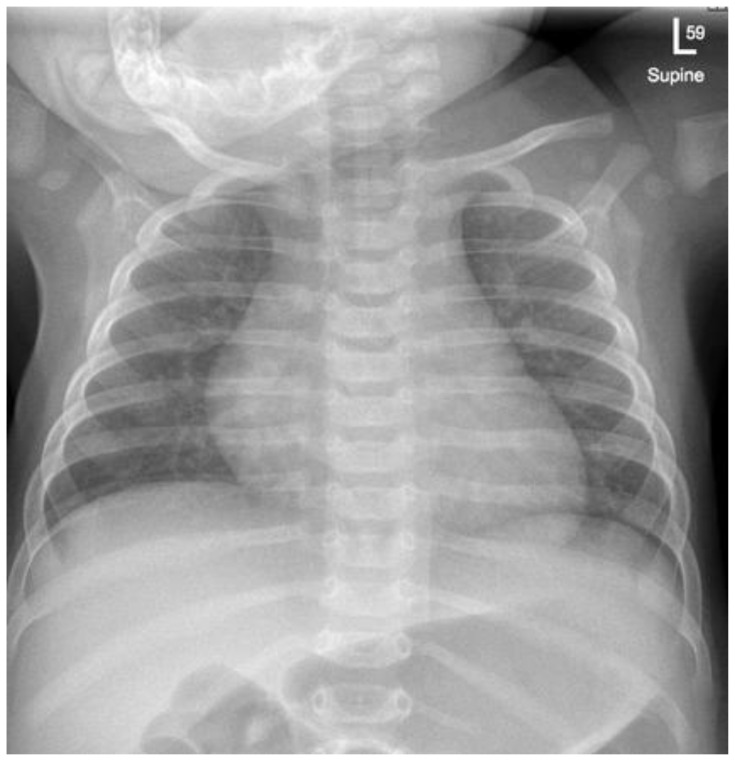


**Figure:**
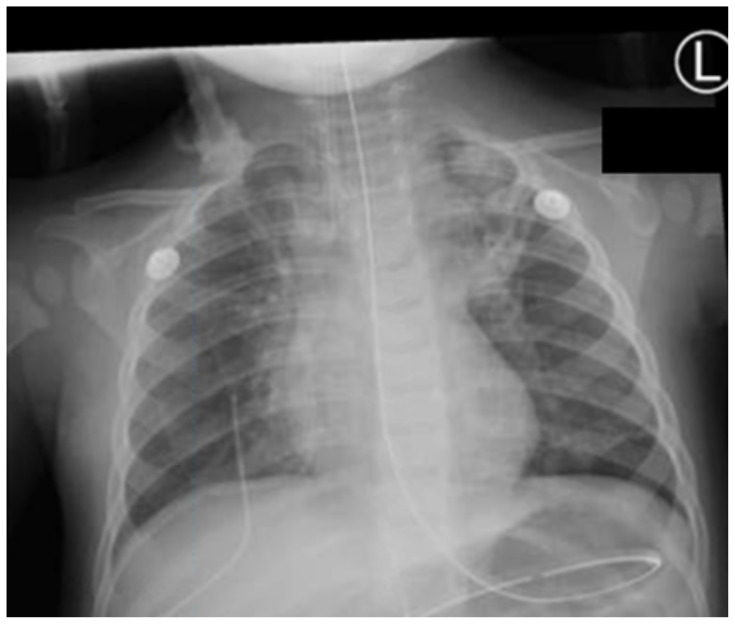


**Figure:**
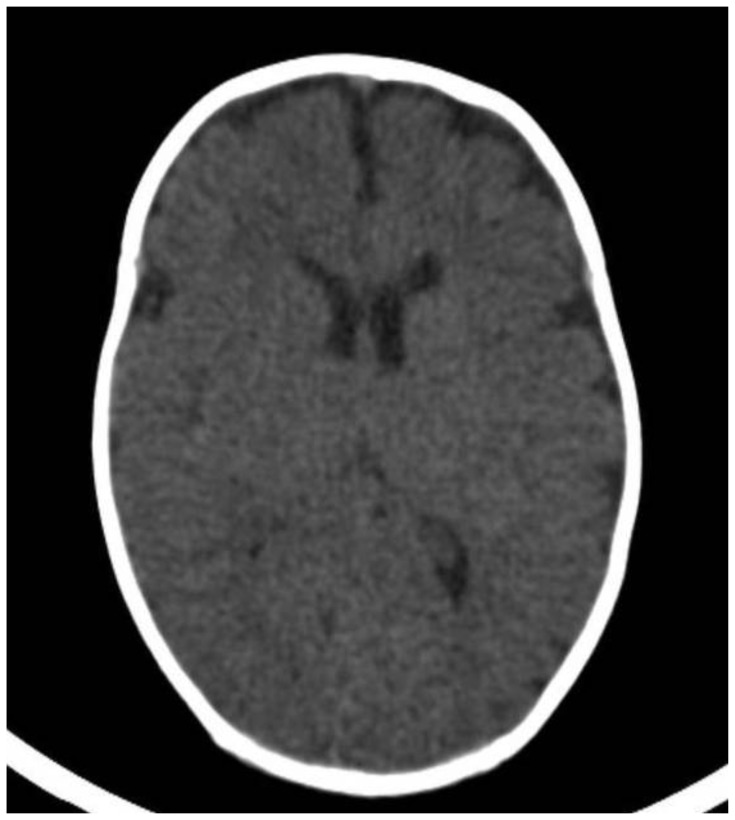


**Table:** 

*CSF Studies*	
WBC	3/mm^3^
RBC	2/mm^3^
Protein	50 mg/dL
Glucose	55 mg/dL

**Table:** SIMULATION EVENTS TABLE:

Minute (state)	Participant action/trigger	Patient status (simulator response) & operator prompts	Monitor display (vital signs)
0:00 (Baseline)	Triage Report Place patient on monitor Learner obtains primary assessment	The patient will have ptosis and the parent will be wiping drool from the infant. Hypotonic manikin setting is used, with ptosis and mydriasis.	T 37°C HR 175 BP 70/50 RR 28 O2 92
	Give Supplemental O2 Peripheral IV, POC Glucose	If no glucose is obtained in the first 5 minutes, nurse prompts, “Do you think we should check a glucose level?”	T 37°C HR 175 BP 70/50 RR 28 O2 92
	Obtain basic history and perform secondary exam	Learners should obtain history including feeding amount and frequency, stooling frequency, and activity level. If no details are obtained, the nurse will prompt with questions for the parent. Nurse prompt: Are you giving anything besides breast feeding? Parent will admit to using honey pacifier to soothe the patient.	T 37°C HR 175 BP 70/50 RR 28 O2 92
	Participants should develop differential and diagnose infant botulism	Sepsis should be considered in the differential diagnosis of this patient, and appropriate work up should be initiated. The decision should be made whether to give antibiotics. Nurse prompt: Should we get blood cultures or give antibiotics?	T 37°C HR 175 BP 70/50 RR 28 O2 92
	Participants should intubate the patient with impending respiratory failure	Patient will have apneic episodes and become more hypoxic and bradycardic. Nurse prompt: “I don’t think this baby is breathing.”	T 37°C HR 130 BP 70/50 RR 20 O2 88
		If participants place supplemental oxygen, NRB, or other noninvasive ventilation, the patient will continue to desaturate with apneic episodes. Nurse prompt: The baby is still not breathing very well.	
	Participants should initiate treatment of botulism	Participants should call poison control or local health department about botulism case. Nurse prompt: “How do we know for sure this is botulism? How is it treated?”	T 37°C HR 160 BP 70/50 RR 40 O2 100
(Case Completion)	Participants should transfer the patient for admission to the intensive care unit (ICU)	The patient should be transferred and admitted to the pediatric intensive care unit for continued management. If participants do not initiate treatment from the ED, then the ICU physician will ask about calling the health department.	

**Figure:**
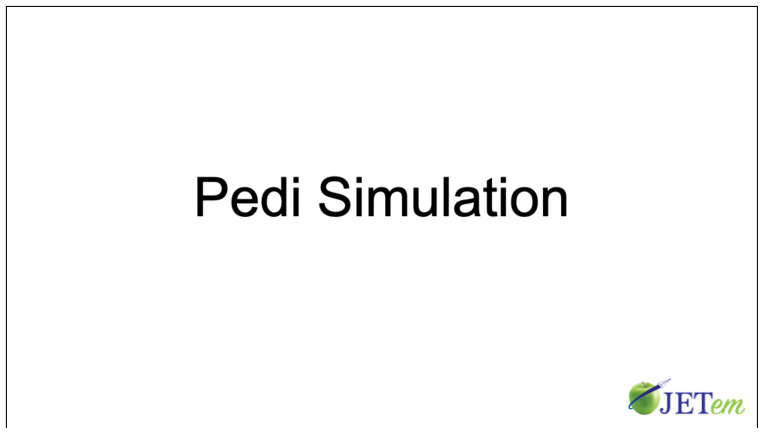


**Table:** 

**Critical Actions:** Place IV, obtain vitals Perform primary survey (airway, breathing, circulation) Obtain history from patient’s parent Recognize need for airway protection, prepare for intubation, perform rapid sequence intubation Communicate necessary information with parent as decisions are made Develop a differential for the hypotonic infant, diagnose infant botulism Mobilize resources to obtain BabyBIG^®^ Transfer the patient for admission to a pediatric intensive care unit	0:00

**Table:** 

	Milestone	Did not achieve level 1	Level 1	Level 2	Level 3
1	**Emergency Stabilization (PC1)**	□ Did not achieve Level 1	□ Recognizes abnormal vital signs	□ Recognizes an unstable patient, requiring intervention Performs primary assessment Discerns data to formulate a diagnostic impression/plan	□ Manages and prioritizes critical actions in a critically ill patient Reassesses after implementing a stabilizing intervention
2	**Performance of focused history and physical (PC2)**	□ Did not achieve Level 1	□ Performs a reliable, comprehensive history and physical exam	□ Performs and communicates a focused history and physical exam based on chief complaint and urgent issues	□ Prioritizes essential components of history and physical exam given dynamic circumstances
3	**Diagnostic studies (PC3)**	□ Did not achieve Level 1	□ Determines the necessity of diagnostic studies	□ Orders appropriate diagnostic studies. Performs appropriate bedside diagnostic studies/procedures	□ Prioritizes essential testing Interprets results of diagnostic studies Reviews risks, benefits, contraindications, and alternatives to a diagnostic study or procedure
4	**Diagnosis (PC4)**	□ Did not achieve Level 1	□ Considers a list of potential diagnoses	□ Considers an appropriate list of potential diagnosis May or may not make correct diagnosis	□ Makes the appropriate diagnosis Considers other potential diagnoses, avoiding premature closure
5	**Pharmacotherapy (PC5)**	□ Did not achieve Level 1	□ Asks patient for drug allergies	□ Selects an medication for therapeutic intervention, consider potential adverse effects	□ Selects the most appropriate medication and understands mechanism of action, effect, and potential side effects Considers and recognizes drug-drug interactions
6	**Observation and reassessment (PC6)**	□ Did not achieve Level 1	□ Reevaluates patient at least one time during case	□ Reevaluates patient after most therapeutic interventions	□ Consistently evaluates the effectiveness of therapies at appropriate intervals
7	**Disposition (PC7)**	□ Did not achieve Level 1	□ Appropriately selects whether to admit or discharge the patient	□ Appropriately selects whether to admit or discharge Involves the expertise of some of the appropriate specialists	□ Educates the patient appropriately about their disposition Assigns patient to an appropriate level of care (ICU/Tele/Floor) Involves expertise of all appropriate specialists
9	**General Approach to Procedures (PC9)**	□ Did not achieve Level 1	□ Identifies pertinent anatomy and physiology for a procedure Uses appropriate Universal Precautions	□ Obtains informed consent Knows indications, contraindications, anatomic landmarks, equipment, anesthetic and procedural technique, and potential complications for common ED procedures	□ Determines a back-up strategy if initial attempts are unsuccessful Correctly interprets results of diagnostic procedure
20	**Professional Values (PROF1)**	□ Did not achieve Level 1	□ Demonstrates caring, honest behavior	□ Exhibits compassion, respect, sensitivity and responsiveness	□ Develops alternative care plans when patients’ personal beliefs and decisions preclude standard care
22	**Patient centered communication (ICS1)**	□ Did not achieve level 1	□ Establishes rapport and demonstrates empathy to patient (and family) Listens effectively	□ Elicits patient’s reason for seeking health care	□ Manages patient expectations in a manner that minimizes potential for stress, conflict, and misunderstanding. Effectively communicates with vulnerable populations, (at risk patients and families)
23	**Team management (ICS2)**	□ Did not achieve level 1	□ Recognizes other members of the patient care team during case (nurse, techs)	□ Communicates pertinent information to other healthcare colleagues	□ Communicates a clear, succinct, and appropriate handoff with specialists and other colleagues Communicates effectively with ancillary staff

**Table 1:** Post Simulation Survey

Question	Response
This experience will improve my performance in an actual clinical setting.	Likert
This simulation was a valuable learning experience.	Likert
The debriefing was a valuable learning experience.	Likert
How could this experience be improved?	Free Response
What were the strengths of this experience?	Free Response

**Table 2:** Open ended feedback

***“Good expansive differential for infantile decreased tone”***
***“Rare case with a large ddx to consider”***
***“Lots of things I was unaware of including baby big only available in CA, lots of useful info”***
***“Learned a lot about hypoglycemia treatment in peds as well as botulism treatment”***
***“Good pathology, good workup, good differentials”***

## INSTRUCTOR MATERIALS

**Case 1 Title:** Infant Botulism

**Case Description & Diagnosis (short synopsis):** The patient is a 2-month-old male with no past medical history or significant birth history presenting to a community emergency department with hypotonia, decreased oral intake, drooling, and constipation. He presents to the emergency department with his parent. The parent states that prior to this presentation, the patient had been fussy and colicky and if asked, will admit they gave him a honey pacifier to soothe him. The learner should perform a primary survey to assess the patient. The learner should recognize significant secretions and hypotonia and the need to protect the patient’s airway. The goal of this case is to recognize infant botulism as a rare cause of hypotonia in infants and mobilize appropriate resources to provide treatment. Learners will care for a critically ill infant and transfer them to the PICU.

**Equipment or Props Needed:**

High fidelity infant simulation manikin

Water-soluble lubricant jelly to simulate drooling

Cardiac monitor with pulse oximetry

Broselow tape

Intravenous (IV) Supplies

- Intraosseous (IO) Needle and Drill
- Angiocatheter
- IV tubing
- Crystalloid IV fluids

Airway Adjuncts

- Nasal cannula
- Non-rebreather mask
- Bag-valve-mask
- BiPAP mask
- Video Laryngoscope
- Direct Laryngoscope
- Endotracheal tube with stylet
- Laryngeal mask airway

Medications

- Ketamine
- Rocuronium
- Succinylcholine
- Dextrose 10%
- Fentanyl
- Midazolam
- Propofol
- Etomidate

**Confederates needed:**

- Parent of infant (with script)
- Nurse (with script and prompts)

**Stimulus Inventory:**

**Background and brief information:** The scenario takes place in a community emergency department. A parent brings their 2-month-old infant with a chief complaint of poor feeding.

**Initial presentation:** The patient has come from home by private vehicle and has been triaged by nursing as an ESI (emergency severity index) level 2. The patient is a 2-month-old male with no past medical history or significant birth history presenting with hypotonia, decreased oral intake, drooling, and constipation.

**How the scene unfolds:** The patient is a 2-month-old infant who is laying on the gurney in no acute distress. The parent is present and is intermittently wiping drool from the patient’s mouth, simulated by water soluble lubricant. On primary survey, learners should assess the patient’s airway, breathing, and circulation and obtain a full set of vitals. Vital signs are notable for tachycardia, decreased respiratory rate and hypoxia. The learner is expected to place the patient on supplemental oxygen to treat the patient’s hypoxia and use suction to support the airway. Learners should obtain a history from the patient’s parent, including feeding and stooling habits. The parent states that over the past few days their child has had poor feeding and has had decreased number of stools and more difficulty with stooling. Prior to this, the parent states that their baby had been fussy and to soothe him, they gave him a honey pacifier. This information should be given if learners ask about changes in diet or new ingestion in the past week. If history of honey pacifier use is not obtained, then the nurse can prompt for any changes in diet or ingestion. The patient has not taken a full bottle in over 24 hours due to lethargy and has not been waking up for feeds. The parent states that today the infant has not been as active as normal and has been drooling. Learners should perform a physical exam. The physical exam is notable for mydriasis, bilateral ptosis, and hypotonia. The infant mannikin Laerdal SimBaby can simulate ptosis and mydriasis, visual stimulus can be given to replace these findings in low fidelity simulation or high-fidelity pediatric mannequin without these capabilities. The patient will be hypotonic, drooling, with ptosis, and high end-tidal CO_2_. Learners should place an IV and obtain laboratory studies. Learners should obtain a finger-stick blood glucose (FSBG) in the first five minutes and give IV glucose to treat hypoglycemia. If FSBG is not checked, then nursing will prompt learners to check. The learners should recognize the need to intervene on the patient’s airway and perform rapid sequence intubation, regardless of seizure activity. The learners should pick equipment appropriate for the patient’s age, weight, and height and appropriately dose medications. If the parent is not included and updated throughout the resuscitation, then the parent will ask clarifying questions. Once the intubation is performed and the airway is secure, a CT brain and lumbar puncture should be considered as a part of an infectious and altered mental status work-up for this age. Because this case will occur in a community emergency department, learners will transfer the patient to a pediatric center. PICU admission is the only appropriate disposition for this patient. The treatment for botulism, BabyBIG^®^, is only available in California and must be mobilized through the health department. If the health department has not been contacted prior to admission to PICU, the accepting physician will mention obtaining BabyBIG^®^.

**Critical actions:**

1. Place IV, obtain vitals
2. Perform primary survey (airway, breathing, circulation)
3. Obtain history from patient’s parent
4. Recognize need for airway protection, prepare for intubation, perform rapid sequence intubation
5. Communicate necessary interventions with parent as decisions are made
6. Develop a differential for the hypotonic infant, diagnose Infant botulism
7. Mobilize resources to obtain BabyBIG^®^
8. Transfer the patient for admission to a pediatric intensive care unit

**Case Title:** Infant Botulism

**Chief Complaint:** Weakness

**General Appearance:** 2-month-old infant laying on gurney in no acute distress, lethargic.

**Primary Survey:**

- **Airway:** Patient is drooling, parent continues to wipe secretions away from mouth.
- **Breathing:** Bradypnic, shallow breaths, clear to auscultation
- **Circulation:** Tachycardic, 2+ peripheral pulses, symmetrical, capillary refill < 2 sec

**History:**

- **History of present illness:** The patient is a 2-month-old male presenting with hypotonia, decreased oral intake, drooling, and constipation. The parent states that over the past few days their child has had poor feeding and has had decreased number of stools and more difficulty with stooling. Prior to this, the parent states that their baby had been fussy and to soothe him they gave him a honey pacifier. The patient has not taken a full bottle in over 24 hours and has not been waking up for feeds. The parent states that today, the infant has not been as active as normal and has been drooling.
- **Past medical history:** None
- **Past birth history:** Born at term, spontaneous vaginal delivery, no complications
- **Past surgical history:** None
- **Medications:** None
- **Allergies:** No known allergies
- **Social history:** Lives at home with parents
- **Family history:** Non-contributory

**Secondary Survey/Physical Examination:**

- **General appearance:** Well developed infant, lethargic
- **HEENT:**
  ○ **Head:** Normocephalic, atraumatic, fontanelle soft and flat
  ○ **Eyes:** Bilateral ptosis and mydriatic pupils 5mm (programmable for Laerdal SimBaby, can be visual stimulus)
  ○ **Ears:** Within normal limits (wnl)
  ○ **Nose:** wnl
  ○ **Throat:** oropharynx with drooling
- **Neck:** wnl
- **Heart:** Tachycardic, regular rhythm, no murmurs
- **Lungs:** Bradypnic, shallow inspiration, clear to auscultation
- **Abdominal/GI:** Soft, no evidence of pain to palpation, nondistended, bowel sounds diminished
- **Genitourinary:** wnl
- **Rectal:** wnl
- **Extremities:** No deformity or tenderness
- **Back:** wnl
- **Neuro:**
  ○ Down-going plantar reflex, ptosis, decreased facial muscle tone, 1+ reflexes, hypotonic.
  ○ CN (cranial nerve) II: pupil, equal, round, reactive to light
  ○ CN III, IV, VI: bilateral ptosis
  ○ CN VII: poor suck (reported finding by facilitator if asked, unable to demonstrate this on the mannequin)
  ○ CN VIII: Unable to assess secondary to age
  ○ CN IX: uvula midline (reported finding by facilitator if asked, unable to demonstrate this on the mannequin)
  ○ CN X: no gag reflex (reported finding by facilitator if asked, unable to demonstrate this on the mannequin)
  ○ CN XI: Unable to assess secondary to age
  ○ CN XII: Unable to assess secondary to age
- **Skin:** wnl
- **Lymph:** wnl

*Chest X-ray*

Bickle I. Chest Radiograph (pediatric) In: Radiopaedia. https://radiopaedia.org/cases/46490. CC BY-NC-SA 3.0

*Chest X-ray post intubation*

Author’s own image

*CT Brain without contrast*

Glick Y. Normal brain CT - 2-month-old. In Radiopaedia. https://radiopaedia.org/cases/normal-brain-ct-2-month-old-1. CC BY-NC-SA 3.0

## STANDARDIZED PATIENT BRIEFING MATERIALS

### Case: Infant Botulism

#### Nursing Script and Prompts

If point of care glucose is not checked within the first five minutes.

Nurse: “Do you think we should check a glucose level?”

If no details are obtained regarding patient’s feeding and stooling habits, have nurse prompt with questions for the parent:

Nurse: “How often are you feeding, how much?” Parent: “Normally he eats 5 ounces 6–8 times per day. But over the past few days, the amount he is taken in has decreased only taking 1–2 ounces if I can wake him up to feed.”

If history of honey pacifier is not obtained:

Nurse: “Are you giving anything besides breast feeding?” Parent: “I sometimes give honey pacifiers when he is fussy.”

If differential is not stated during case:

Nurse: “What do we think is the diagnosis?”

If blood cultures are not ordered or sepsis is not considered:

Nurse: “Would you like me to draw blood cultures or give antibiotics?”

When the patient becomes apneic, if no intervention is performed:

Nurse: “I don’t think this baby is breathing.”

If the learners provide other supplemental oxygenation besides intubating:

Nurse: “The baby is still not breathing very well; I don’t think it can breathe on its own”:

After intubation, if ventilator settings are not stated:

Nurse: “What ventilator settings should be set?”

If treatment is not initiated for botulism:

Nurse: “How do we know for sure this is botulism? How is it treated?”

#### Parent Script and Prompts

If learners asking about feeding:

Parent: “Normally he eats 5 ounces 6–8 times per day. But over the past few days, the amount he is taken in has decreased only taking 1–2 ounces if I can wake him up to feed.”

If learners asking about stooling:

Parent: “He normally has 4 soft bowel movements a day, but he hasn’t pooped in 2 days.”

If learners ask about urination:

Parent: “He normally has 8 wet diapers a day but I have only had to change him once today.”

If learners ask about any other ingestion or recent changes:

Parent: “I recently started soothing him with these honey pacifiers when he is fussy.”

If learners prepare to intubate without telling parent:

Parent: “Wait what are you doing? Why isn’t he breathing?”

If learners do not tell parent about transfer:

Parent: “When is he going to get a room upstairs?”

If learners do not discuss diagnosis with parent:

Parent: “What is wrong with my baby?”

## OPERATOR MATERIALS

**Diagnosis:**

Infant botulism

**Disposition:**

Admission to the PICU

## DEBRIEFING AND EVALUATION PEARLS

### Infant Botulism

#### Epidemiology

Most cases occur in infants aged 2 to 8 months but can occur as early as 1 week in age and as late as 12 months. Clostridium Botulinum type A and B are the typical species that produce the clinical infant botulism within the United States (US). Of note, type A is seen in the western US and type B is more common in the eastern US. Environmental Clostridium disease occurs in the conditions where spores are found in the soil (Utah, California, and Pennsylvania). Foodborne cases typically occur from ingestion of wild honey or home-canned foods. Once clostridial spores are ingested, they germinate within the gastrointestinal system before neurotoxin is released into the bloodstream to irreversibly bind presynaptic neuromuscular receptors. Botulism causes hypotonia and weakness through the neurotoxin produced. The neurotoxin is taken up into the presynaptic terminals and cleaves the SNARE protein. The SNARE protein is part of fusion complex that allows acetylcholine release into the cleft. The cleaved SNARE protein leads to failed acetylcholine release and flaccid paralysis. Infants are more susceptible to foodborne botulism due to absence of competitive bowel flora.

#### Clinical Features

Botulism is characterized by a descending paralysis. Cranial nerve muscles are affected by weakness first, then trunk, extremities, and diaphragm. In infants, this may present as poor feeding, lethargy, constipation, and progress to hypotonia. As the disease evolves, the patient develops loss of deep tendon reflexes, diminished eye movements/ptosis, decreased gag and suck. The life-threatening stage of disease progression is characterized by respiratory failure and apneic events.

#### Laboratory Studies

Basic laboratory studies will not diagnose botulism; however, laboratory studies will evaluate for other causes of hypotonia and weakness. Clinical suspicion is required to diagnose infant botulism. In most cases of infant botulism, serum samples for botulinum toxin are negative. The most accurate studies for botulism are isolation of C. botulinum spores from the stool and diagnosis is confirmed by botulinum toxin in stool samples. Stool samples can be difficult to obtain due to constipation. Anaerobic cultures take up to 6 days for growth to be seen and toxin detection can take 1–4 days.

#### Electromyographic Findings

EMG can aid in diagnosis; findings include short-duration, low-amplitude motor unit potential.

#### Differential Diagnosis of Hypotonia in Infants

Botulism, SMA (Spinal Muscular Atrophy) type 1, Metabolic disorder (Leigh’s), brainstem encephalitis, stroke, myasthenia gravis, neuromuscular disease, sepsis, drug ingestion, Guillain-Barre, Lambert-Eaton syndrome. The distinguishing feature between botulism and other pathologies causing hypotonia is the history of spore ingestion and the physical exam. Myasthenia gravis in the newborn also presents with hypotonia and bulbar symptoms leading to poor suck and increased secretions; however, ptosis and ocular symptoms are less prevalent. Hypermagnesemia can present with generalized hypotonia; this will be diagnosed by lab results. Guillain-Barre is typically an ascending paralysis with absent reflexes. Sepsis and encephalitis in the newborn will present with fever and preceding infectious symptoms. In SMA Type 1, generalized hypotonia is present, but upper cranial nerves are typically spared, and onset of symptoms is subacute to chronic. The key in diagnosing this case is clinical suspicion and history of botulinum spore ingestion.

#### Treatment

The treatment for infant botulism is intravenous botulism immune globulin (BabyBIG^®^). This can only be obtained from the California Department of Health Services Infant Botulism Treatment and Prevention Program. High suspicion for infant botulism and early mobilization of resources is key because it takes time for immune globulin to be transported to the facility. BabyBIG^®^ binds the toxin and prevents symptoms from progressing and is effective for both type A and type B toxins. BabyBIG^®^ should be given prior to lab confirmation of botulism. However, it does not reverse current symptoms, so administration should be done as soon as possible. BabyBIG^®^ administration reduced mean hospital stay (2.6 weeks vs 5.7 weeks), reduced mean ICU stay, and has no serious adverse effects. A single dose will neutralize all C. botulinum toxin available for absorption in the body for at least 6 months. Supportive care is the mainstay of treatment in these patients. Most patients require intensive care and half require mechanical ventilation for respiratory failure. Of note, antibiotics are not recommended due to concern for lysis and increased toxin burden.

Treatment for hypoglycemia in infants depends on ability to swallow and IV access. If the patient is conscious and able to swallow safely, hypoglycemia can be treated with glucose gel, sweetened juice 0.3 g/kg goal of 10–20 g. If the patient is altered or unable to swallow, then IV glucose should be given. The goal dosage is 0.5 g/kg and volume and concentration depend on the patient’s age. For infants and young children less than 5 years of age, 5 mL/kg of 10% dextrose solution (D10) should be administered. For patient’s older than 5 years old, 2mL/kg of 25% dextrose solution (D25) should be administered. If the patient is unable to take oral glucose and IV access is unable to be obtained, consider IO insertion for access. However, glucagon can be given to treat hypoglycemia. The dosing is 0.5 mg for patients that weigh less than 25 kg and 1 mg for patients weighing greater than 25 kg.

Rocuronium is the drug of choice for RSI in patients with botulism because the paralytic is non-depolarizing. Use of succinylcholine can cause lethal hyperkalemia in patients with botulism, due to depolarization. Impaired acetylcholine release due to botulism toxin leads to upregulation of acetylcholine receptors and subsequent depolarization leads to potassium efflux and hyperkalemia.

Intubation equipment size is important to consider in pediatric patients. Broselow tape can be used to select equipment size and dosing of common resuscitation medications based on height. A two-month-old should be intubated using a Miller 1 blade. The formula for endotracheal tube (ETT) size is age/4+4 for uncuffed tubes and age/4+3.5 for cuffed tubes. For this patient, a 3.5 cuffed ETT should be used and inserted to a depth of 10–11 cm, the size of the ETT multiplied by 3. Post intubation ventilator settings are particularly important in the community emergency department setting. Respiratory therapists with or without pediatric training may not be available to assist. Volume control is appropriate in this setting and the tidal volume should be set based on weight 6–8 cc/kg. Respiratory rate should be set within the normal range for this patient’s age: 30–60 breaths per minute. Positive end-expiratory pressure (PEEP) of 5 cm of water is needed to prevent atelectasis and FiO2 can be titrated based on O_2_Sat.

#### Other debriefing points

- At what point do you initiate the transfer process in a sick infant while in a community ED (specifically: do you wait for the anti-toxin to arrive?)
- Discuss how learners felt regarding communication amongst the participants, especially if there is confusion regarding the diagnosis.
- Discuss how learners felt communicating with the parent of the child regarding diagnosis and treatment.
- If the hospital has a protocol for botulism, the instructors should be prepared to discuss this protocol.

## SIMULATION ASSESSMENT

*Case: Infant Botulism*

Learner: _________________________________________

***Assessment Timeline***

This timeline is to help observers assess their learners. It allows observer to make notes on when learners performed various tasks, which can help guide debriefing discussion.

**Critical Actions:**

□ Place IV, obtain vitals
□ Perform primary survey (airway, breathing, circulation)
□ Obtain history from patient’s parent
□ Recognize need for airway protection, prepare for intubation, perform rapid sequence intubation
□ Communicate necessary information with parent as decisions are made
□ Develop a differential for the hypotonic infant, diagnose infant botulism
□ Mobilize resources to obtain BabyBIG^®^
□ Transfer the patient for admission to a pediatric intensive care unit

**Summative and formative comments:**

**Milestones assessment:**
