# Supplementary material for: Infant Botulism
Source: J Educ Teach Emerg Med. 2022 Apr 15;7(2):S48–77. doi: 10.21980/J8X35W (PMC10332751; doi:10.21980/J8X35W)
Supplement: Supplementary file 1 [file JETem-7-2-S48-supp1.pptx]

## Slide 1
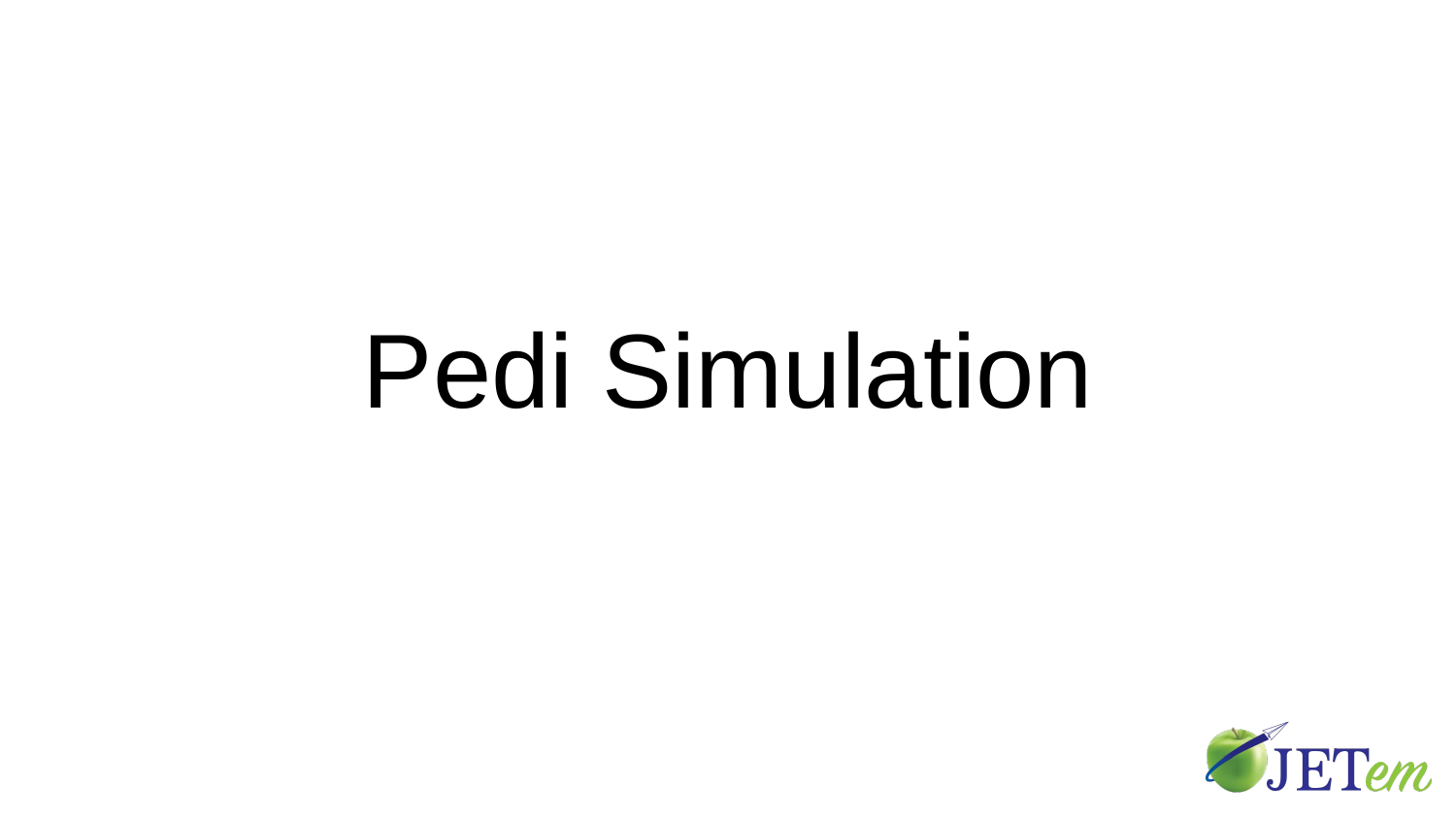

# Pedi Simulation

## Slide 2
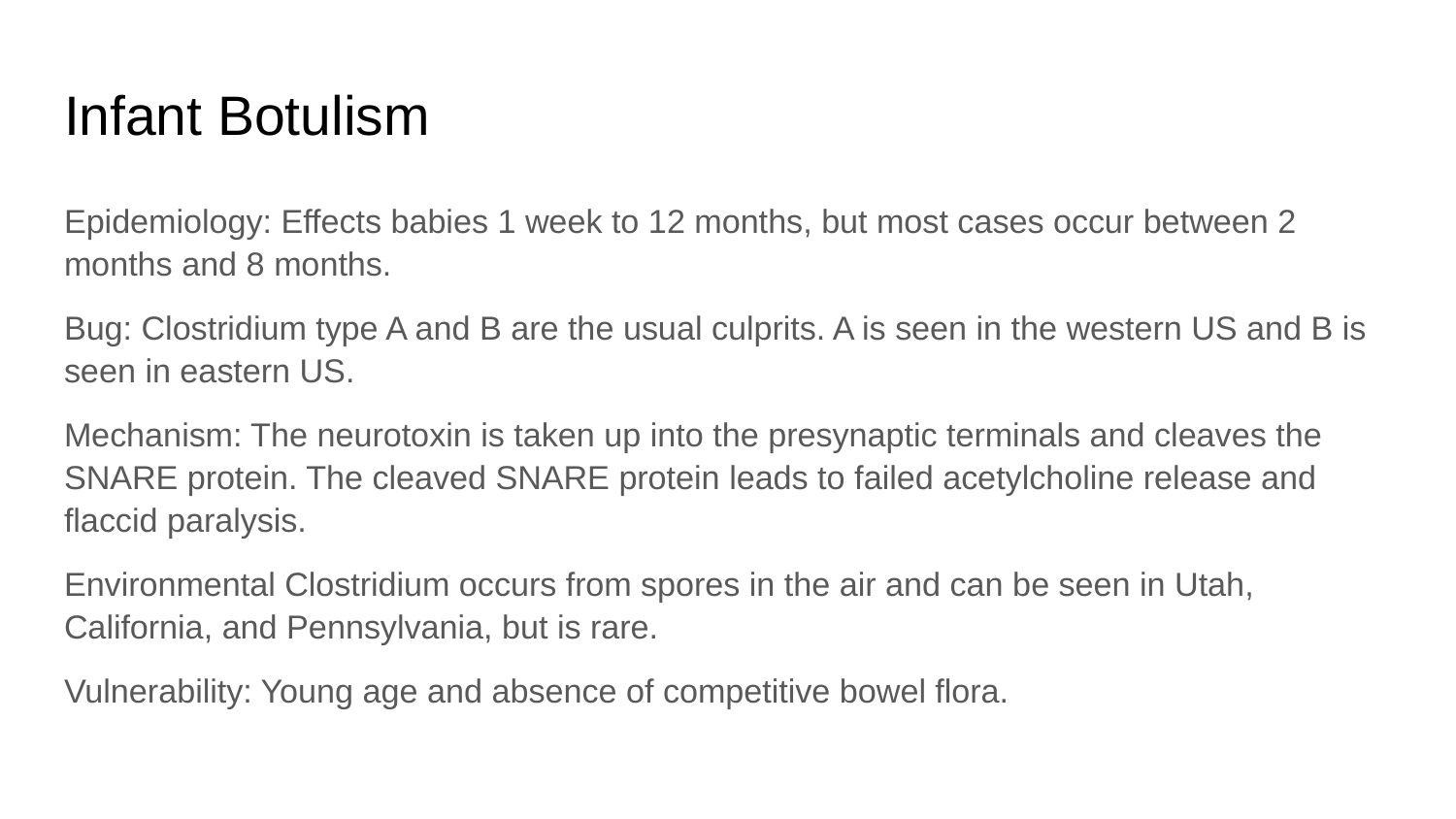

# Infant Botulism
Epidemiology: Effects babies 1 week to 12 months, but most cases occur between 2 months and 8 months.
Bug: Clostridium type A and B are the usual culprits. A is seen in the western US and B is seen in eastern US.
Mechanism: The neurotoxin is taken up into the presynaptic terminals and cleaves the SNARE protein. The cleaved SNARE protein leads to failed acetylcholine release and flaccid paralysis.
Environmental Clostridium occurs from spores in the air and can be seen in Utah, California, and Pennsylvania, but is rare.
Vulnerability: Young age and absence of competitive bowel flora.

## Slide 3
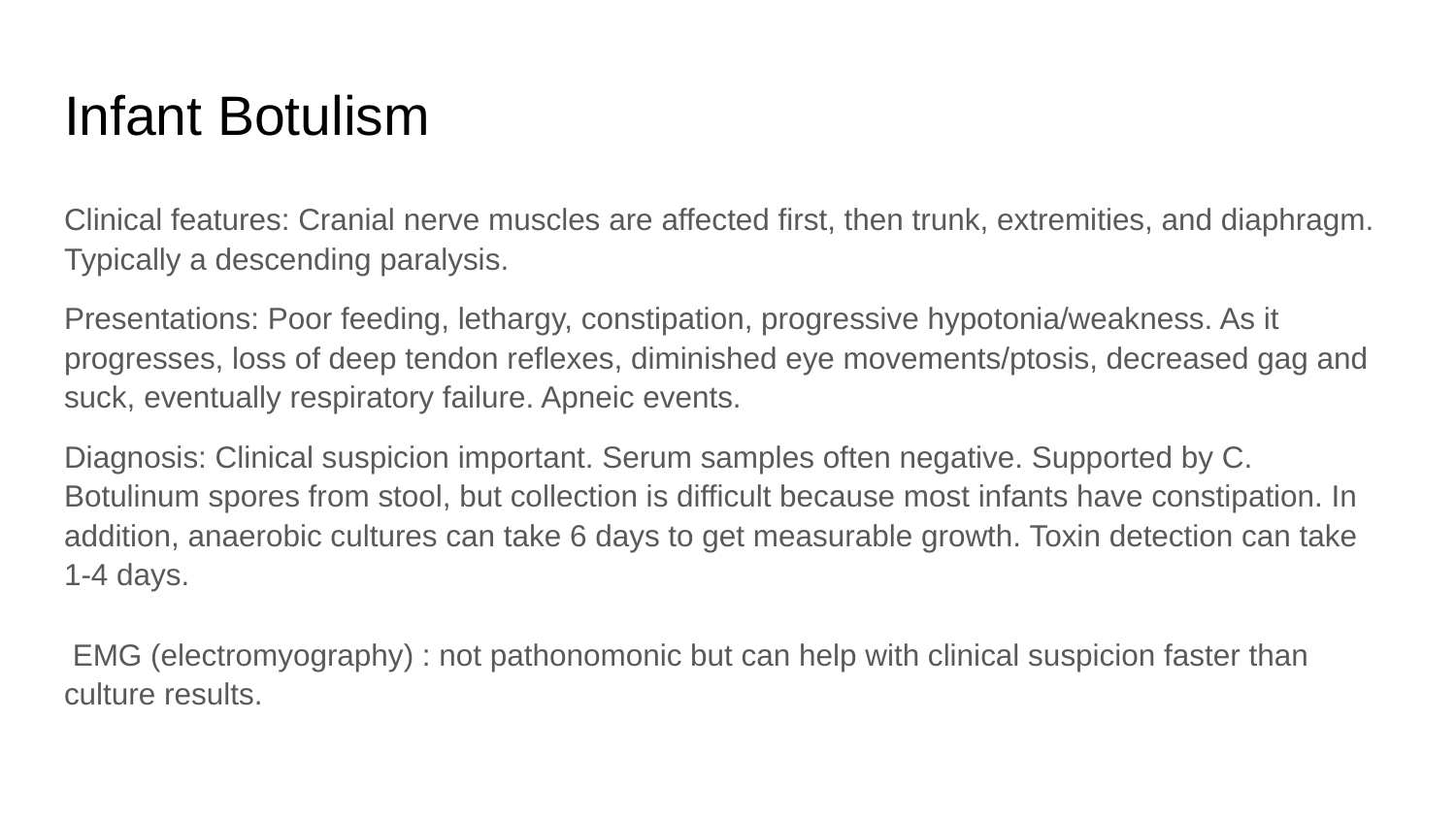

# Infant Botulism
Clinical features: Cranial nerve muscles are affected first, then trunk, extremities, and diaphragm. Typically a descending paralysis.
Presentations: Poor feeding, lethargy, constipation, progressive hypotonia/weakness. As it progresses, loss of deep tendon reflexes, diminished eye movements/ptosis, decreased gag and suck, eventually respiratory failure. Apneic events.
Diagnosis: Clinical suspicion important. Serum samples often negative. Supported by C. Botulinum spores from stool, but collection is difficult because most infants have constipation. In addition, anaerobic cultures can take 6 days to get measurable growth. Toxin detection can take 1-4 days.
 EMG (electromyography) : not pathonomonic but can help with clinical suspicion faster than culture results.

## Slide 4
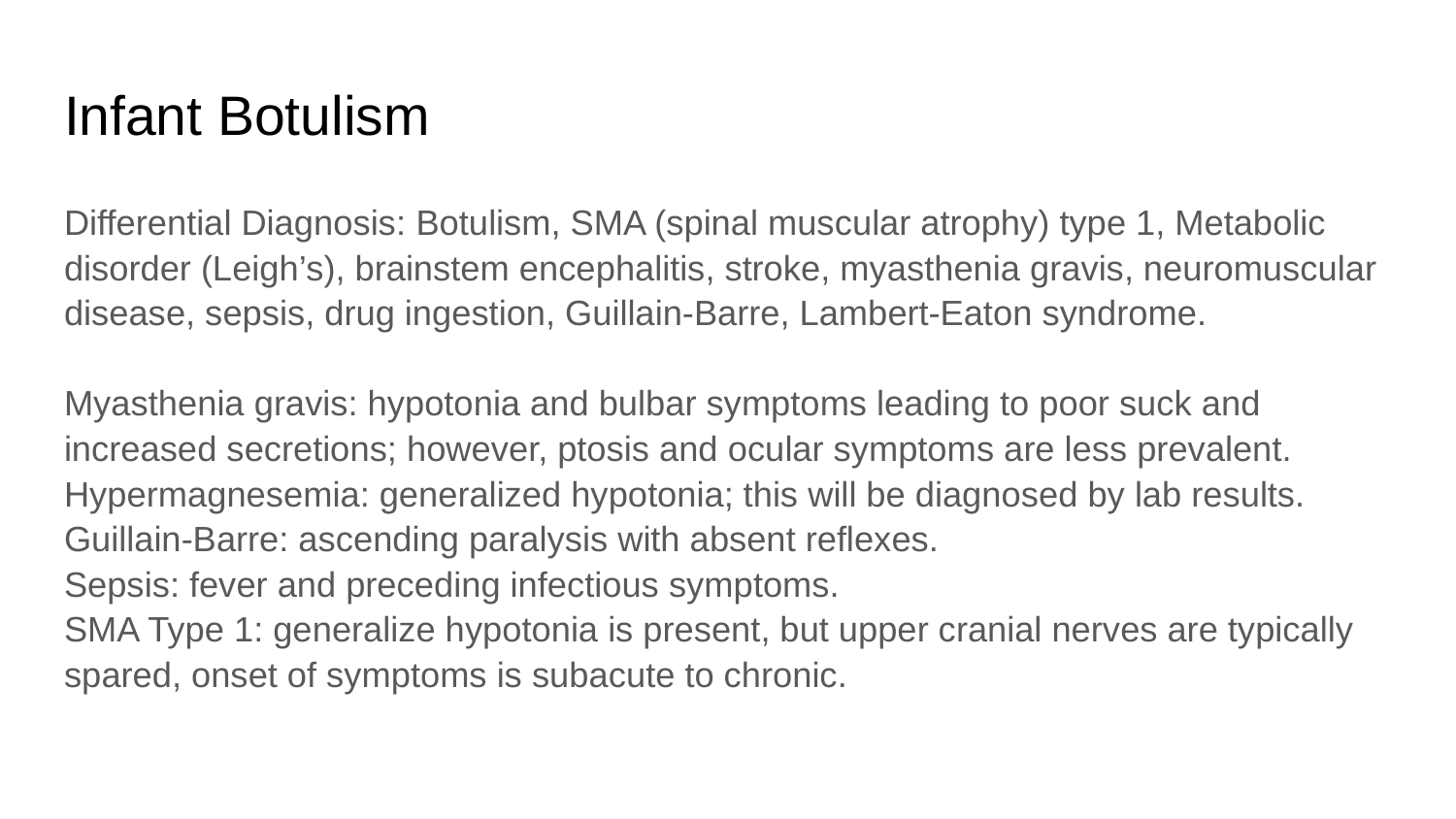

# Infant Botulism
Differential Diagnosis: Botulism, SMA (spinal muscular atrophy) type 1, Metabolic disorder (Leigh’s), brainstem encephalitis, stroke, myasthenia gravis, neuromuscular disease, sepsis, drug ingestion, Guillain-Barre, Lambert-Eaton syndrome.
Myasthenia gravis: hypotonia and bulbar symptoms leading to poor suck and increased secretions; however, ptosis and ocular symptoms are less prevalent. Hypermagnesemia: generalized hypotonia; this will be diagnosed by lab results. Guillain-Barre: ascending paralysis with absent reflexes.
Sepsis: fever and preceding infectious symptoms.
SMA Type 1: generalize hypotonia is present, but upper cranial nerves are typically spared, onset of symptoms is subacute to chronic.

## Slide 5
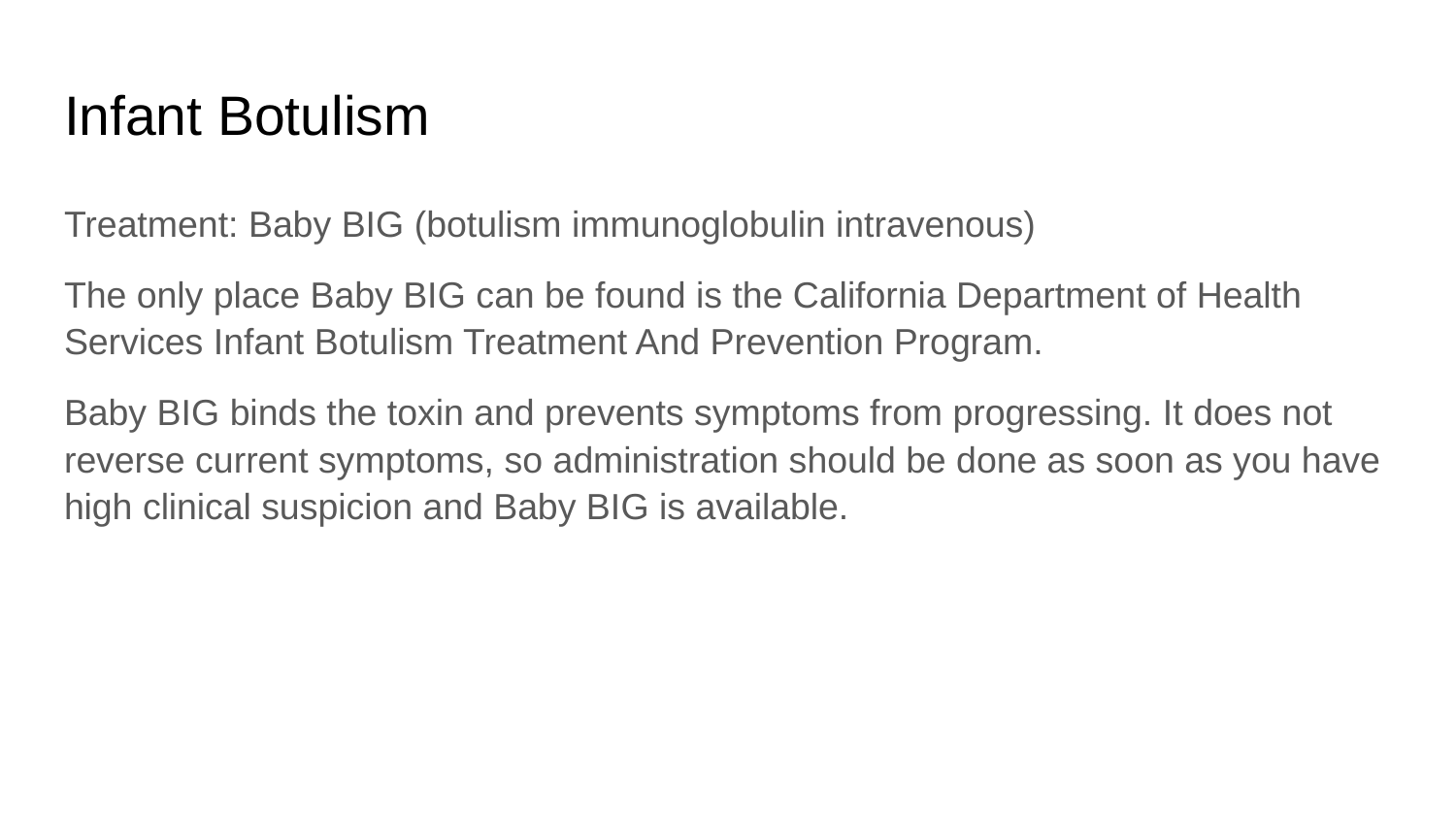

# Infant Botulism
Treatment: Baby BIG (botulism immunoglobulin intravenous)
The only place Baby BIG can be found is the California Department of Health Services Infant Botulism Treatment And Prevention Program.
Baby BIG binds the toxin and prevents symptoms from progressing. It does not reverse current symptoms, so administration should be done as soon as you have high clinical suspicion and Baby BIG is available.

## Slide 6
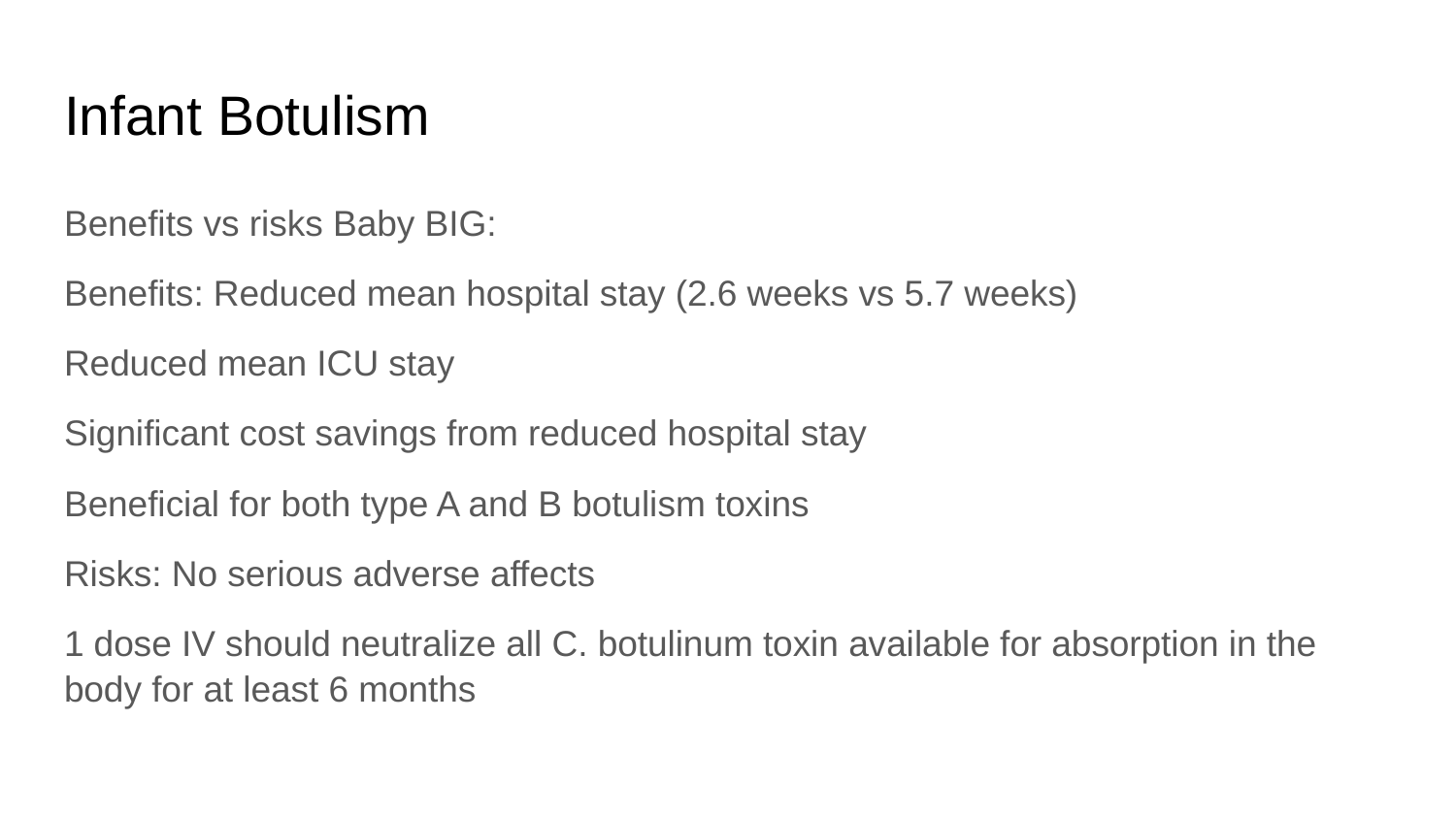

# Infant Botulism
Benefits vs risks Baby BIG:
Benefits: Reduced mean hospital stay (2.6 weeks vs 5.7 weeks)
Reduced mean ICU stay
Significant cost savings from reduced hospital stay
Beneficial for both type A and B botulism toxins
Risks: No serious adverse affects
1 dose IV should neutralize all C. botulinum toxin available for absorption in the body for at least 6 months

## Slide 7
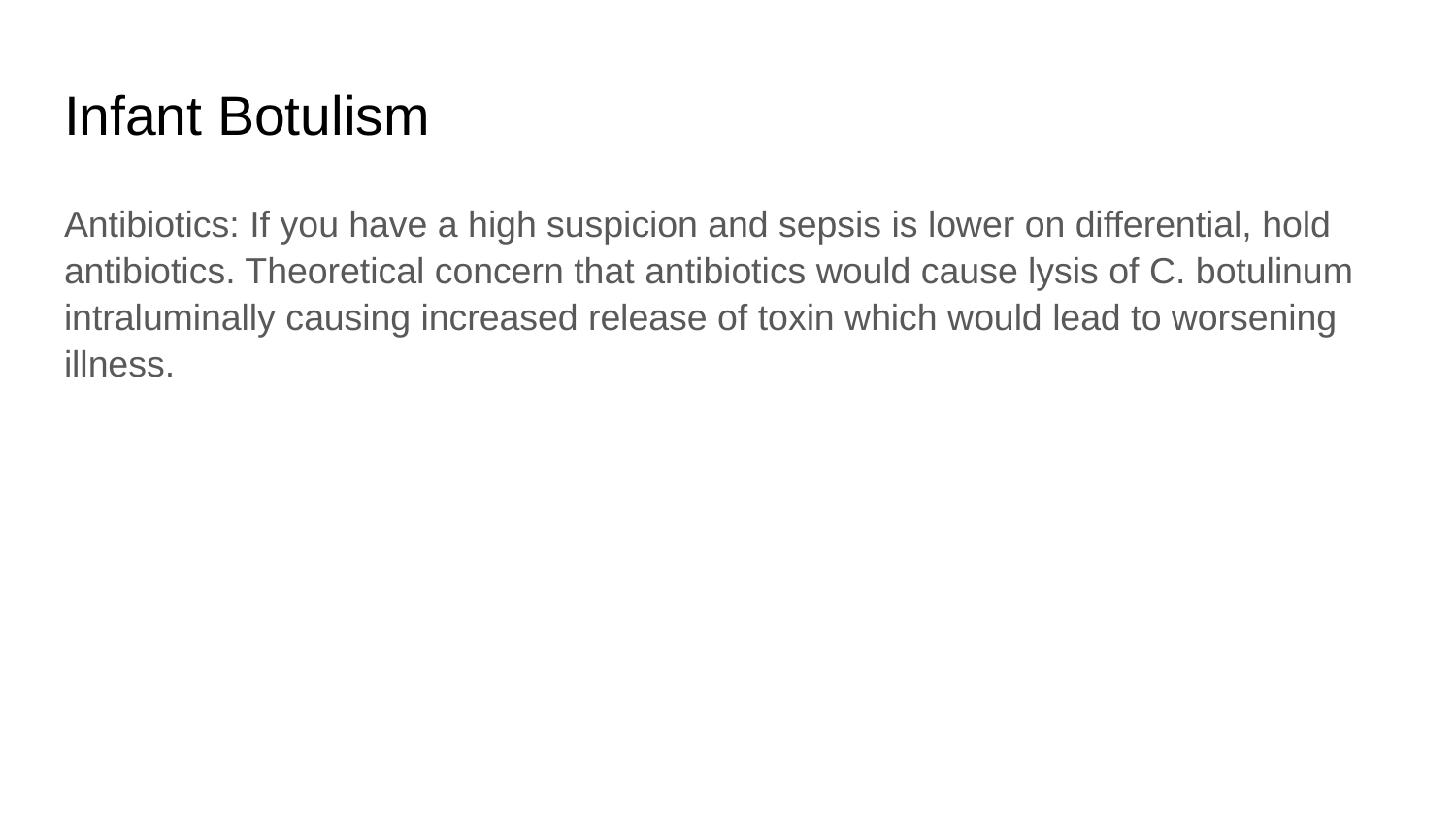

# Infant Botulism
Antibiotics: If you have a high suspicion and sepsis is lower on differential, hold antibiotics. Theoretical concern that antibiotics would cause lysis of C. botulinum intraluminally causing increased release of toxin which would lead to worsening illness.

## Slide 8
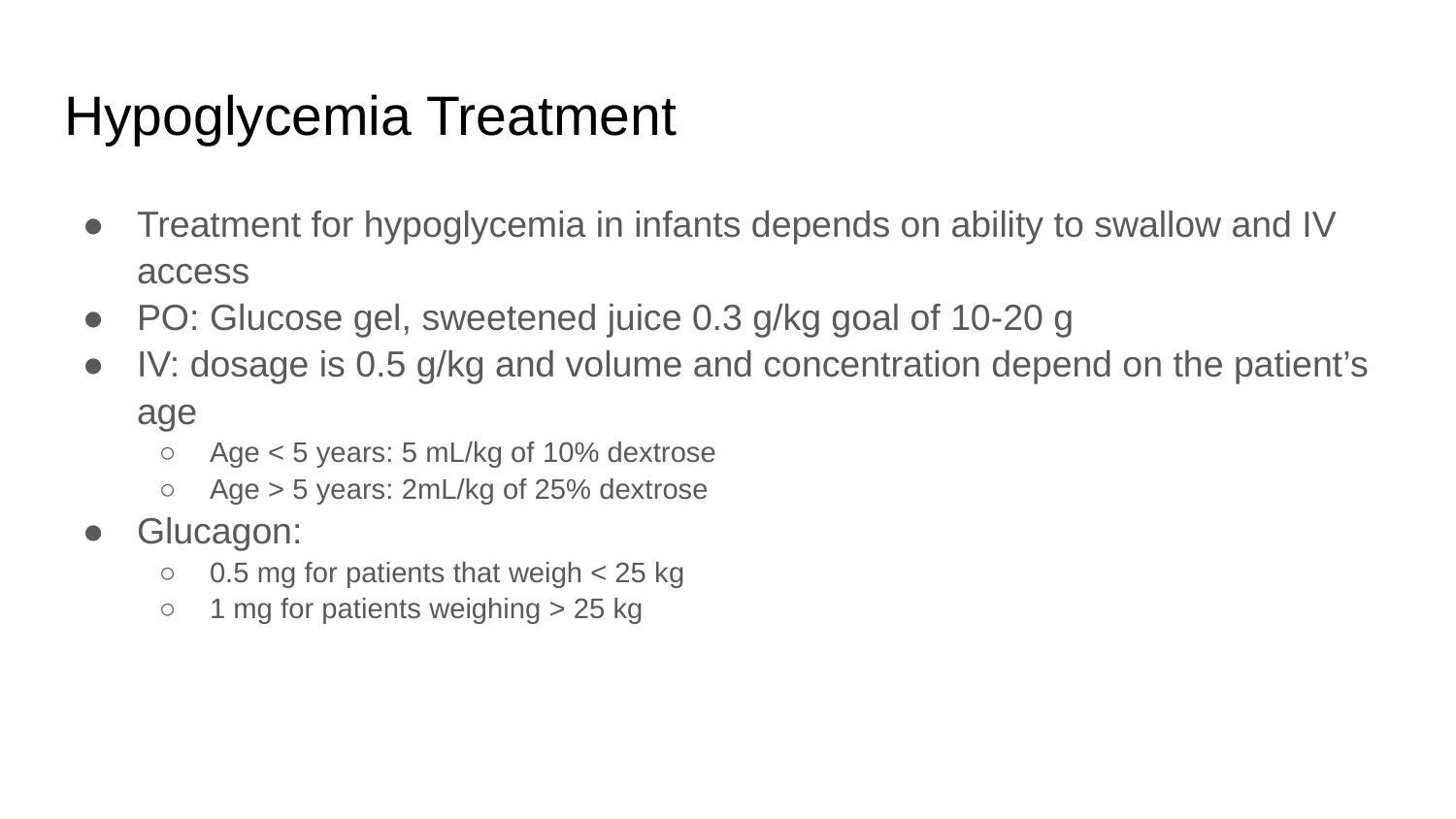

# Hypoglycemia Treatment
Treatment for hypoglycemia in infants depends on ability to swallow and IV access
PO: Glucose gel, sweetened juice 0.3 g/kg goal of 10-20 g
IV: dosage is 0.5 g/kg and volume and concentration depend on the patient’s age
Age < 5 years: 5 mL/kg of 10% dextrose
Age > 5 years: 2mL/kg of 25% dextrose
Glucagon:
0.5 mg for patients that weigh < 25 kg
1 mg for patients weighing > 25 kg

## Slide 9
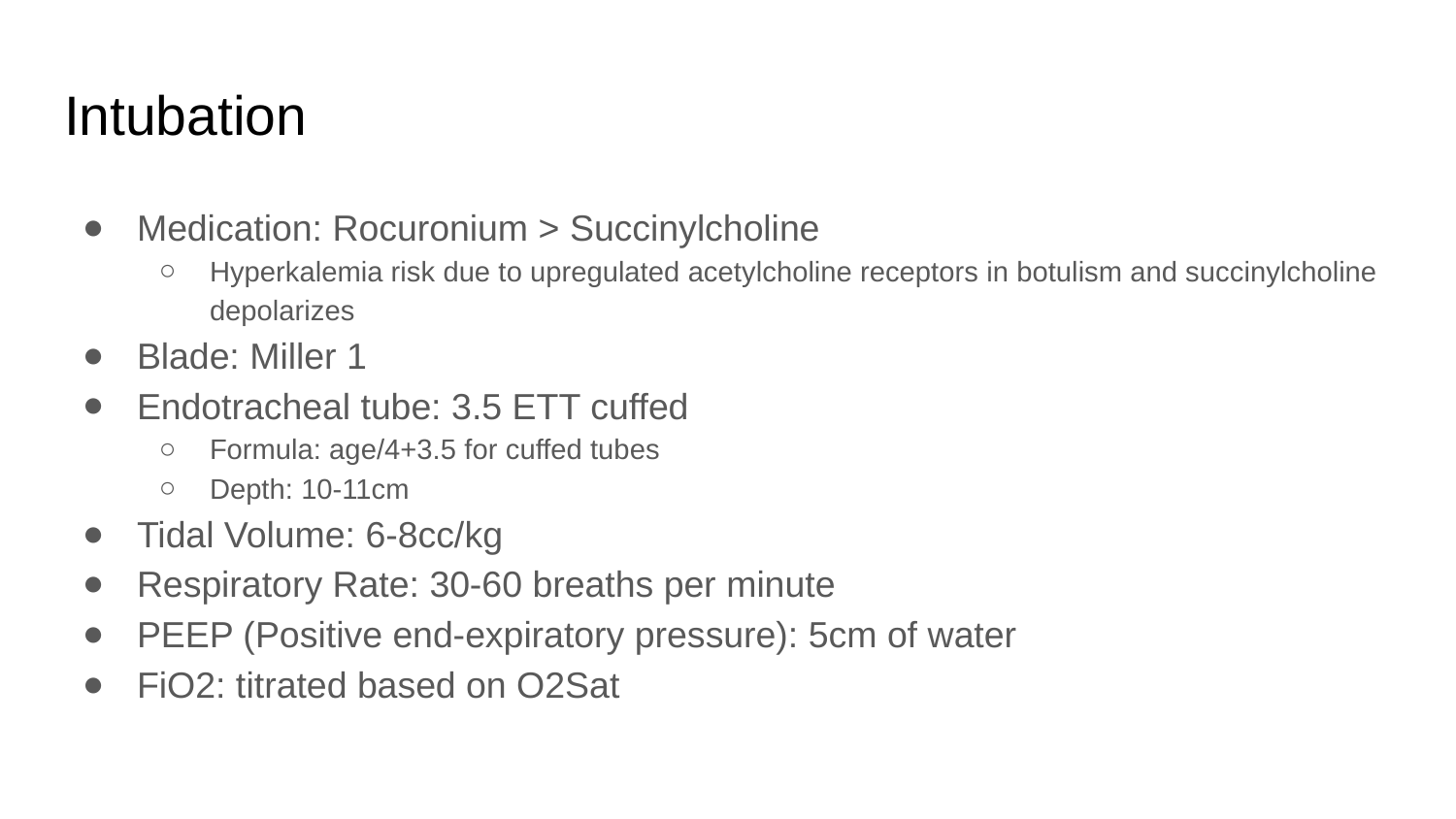

# Intubation
Medication: Rocuronium > Succinylcholine
Hyperkalemia risk due to upregulated acetylcholine receptors in botulism and succinylcholine depolarizes
Blade: Miller 1
Endotracheal tube: 3.5 ETT cuffed
Formula: age/4+3.5 for cuffed tubes
Depth: 10-11cm
Tidal Volume: 6-8cc/kg
Respiratory Rate: 30-60 breaths per minute
PEEP (Positive end-expiratory pressure): 5cm of water
FiO2: titrated based on O2Sat

## Slide 10
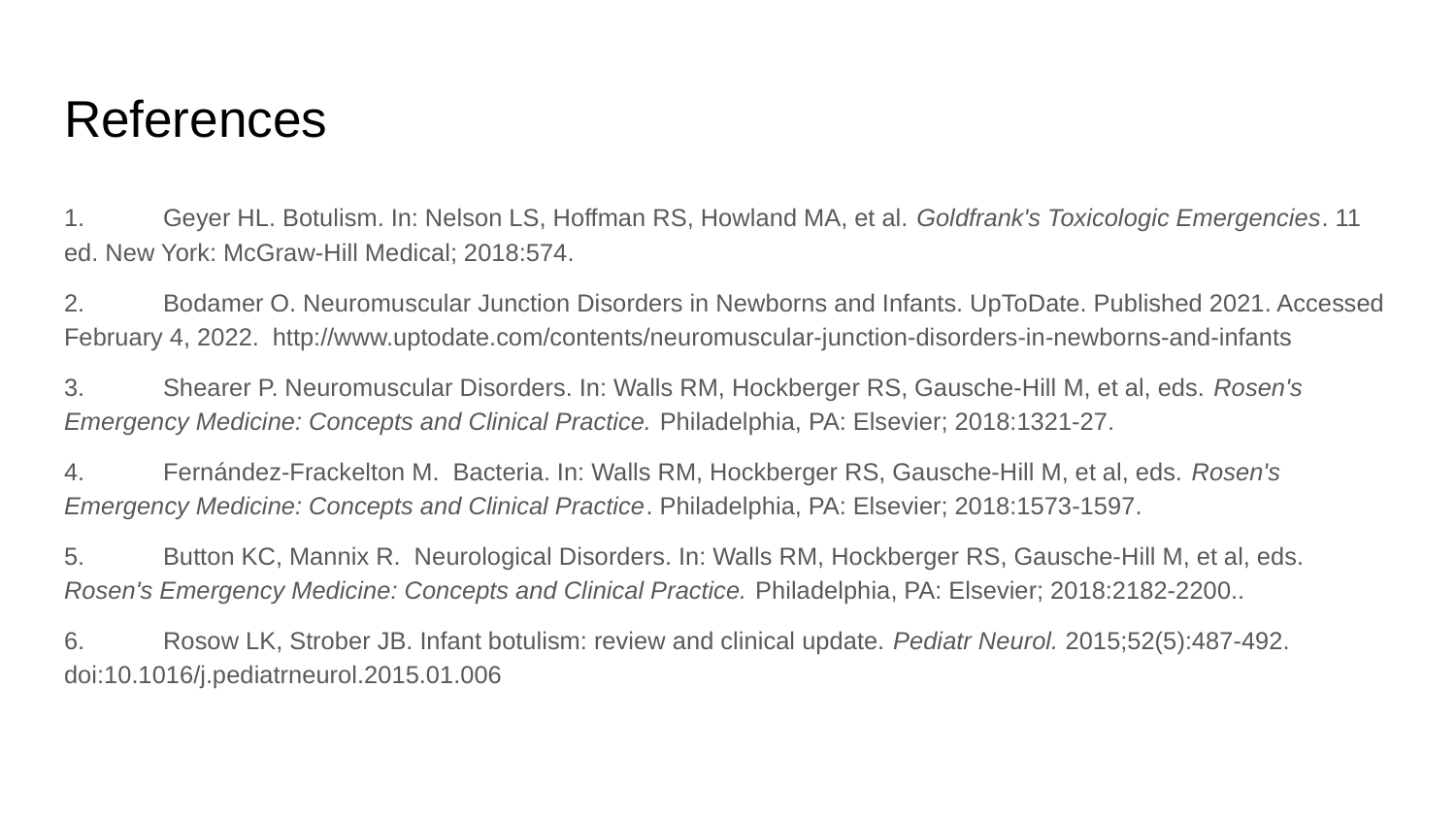

# References
1.	Geyer HL. Botulism. In: Nelson LS, Hoffman RS, Howland MA, et al. Goldfrank's Toxicologic Emergencies. 11 ed. New York: McGraw-Hill Medical; 2018:574.
2.	Bodamer O. Neuromuscular Junction Disorders in Newborns and Infants. UpToDate. Published 2021. Accessed February 4, 2022. http://www.uptodate.com/contents/neuromuscular-junction-disorders-in-newborns-and-infants
3.	Shearer P. Neuromuscular Disorders. In: Walls RM, Hockberger RS, Gausche-Hill M, et al, eds. Rosen's Emergency Medicine: Concepts and Clinical Practice. Philadelphia, PA: Elsevier; 2018:1321-27.
4.	Fernández-Frackelton M. Bacteria. In: Walls RM, Hockberger RS, Gausche-Hill M, et al, eds. Rosen's Emergency Medicine: Concepts and Clinical Practice. Philadelphia, PA: Elsevier; 2018:1573-1597.
5.	Button KC, Mannix R. Neurological Disorders. In: Walls RM, Hockberger RS, Gausche-Hill M, et al, eds. Rosen's Emergency Medicine: Concepts and Clinical Practice. Philadelphia, PA: Elsevier; 2018:2182-2200..
6.	Rosow LK, Strober JB. Infant botulism: review and clinical update. Pediatr Neurol. 2015;52(5):487-492. doi:10.1016/j.pediatrneurol.2015.01.006
