# Supplementary material for: Infant Botulism
Source: J Educ Teach Emerg Med. 2022 Apr 15;7(2):S48–77. doi: 10.21980/J8X35W (PMC10332751; doi:10.21980/J8X35W)
Supplement: Supplementary file 2 [file JETem-7-2-S48-supp2.pptx]

## Slide 1
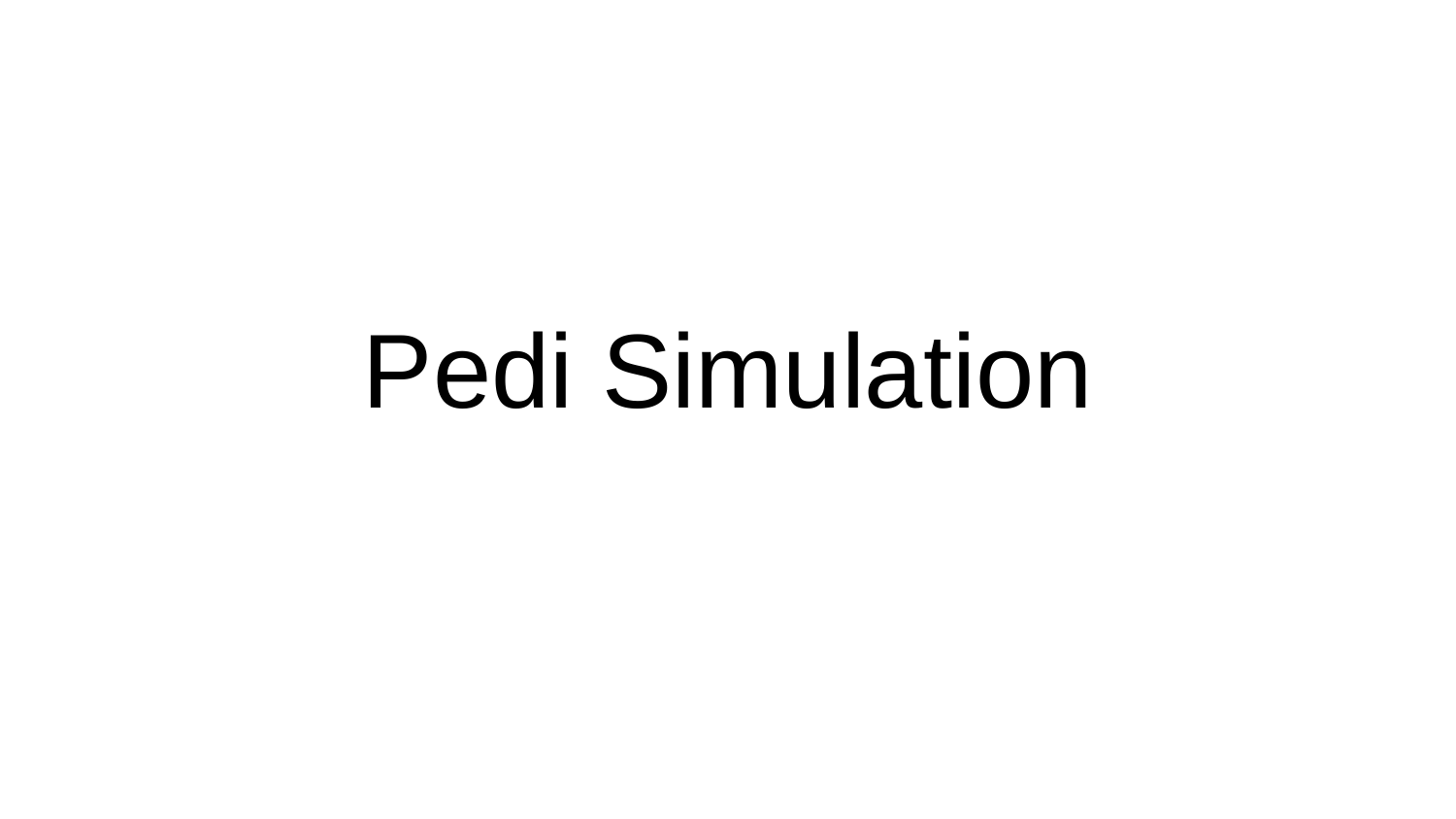

# Pedi Simulation

## Slide 2
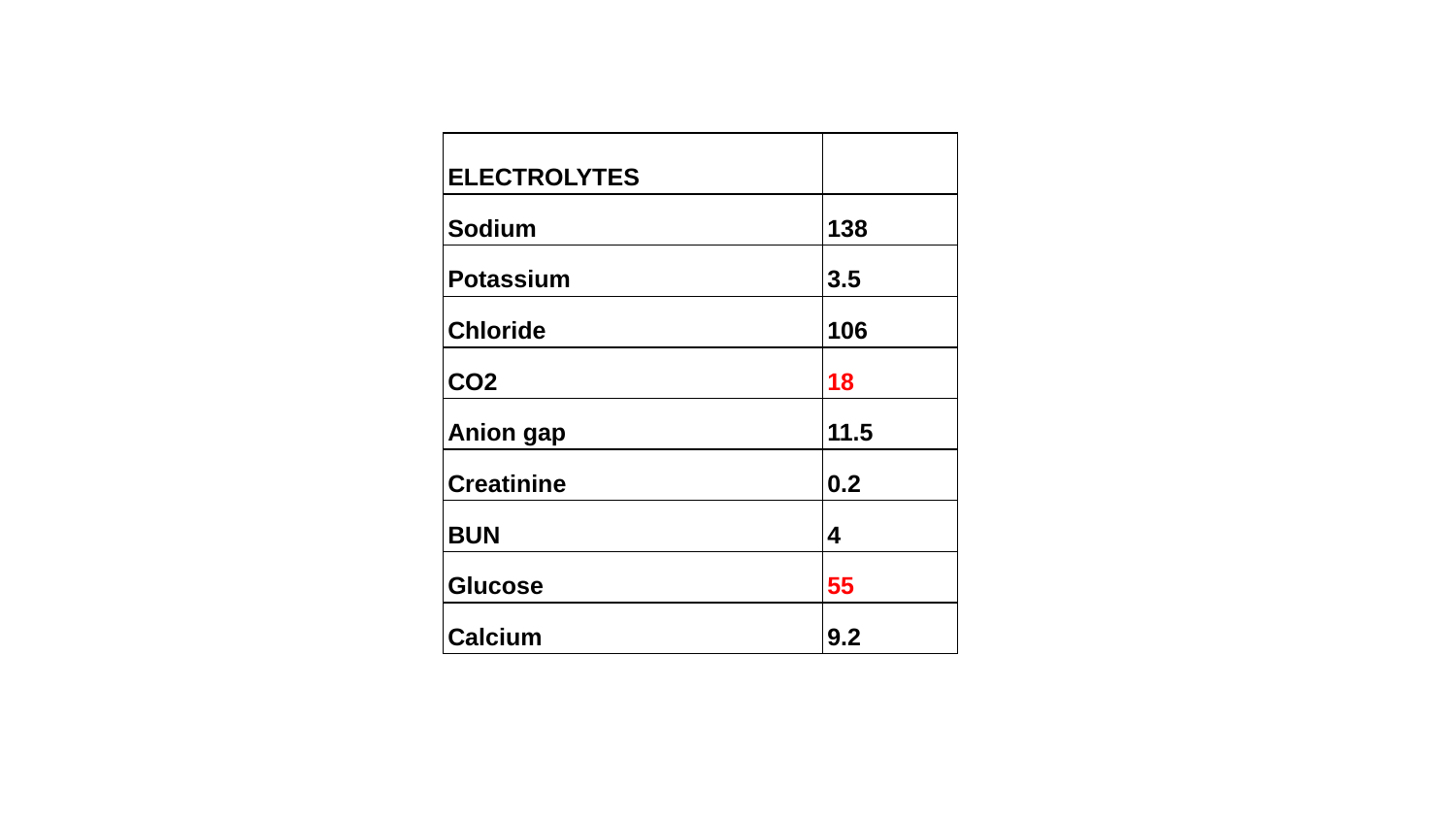

#
| ELECTROLYTES | |
| --- | --- |
| Sodium | 138 |
| Potassium | 3.5 |
| Chloride | 106 |
| CO2 | 18 |
| Anion gap | 11.5 |
| Creatinine | 0.2 |
| BUN | 4 |
| Glucose | 55 |
| Calcium | 9.2 |

## Slide 3
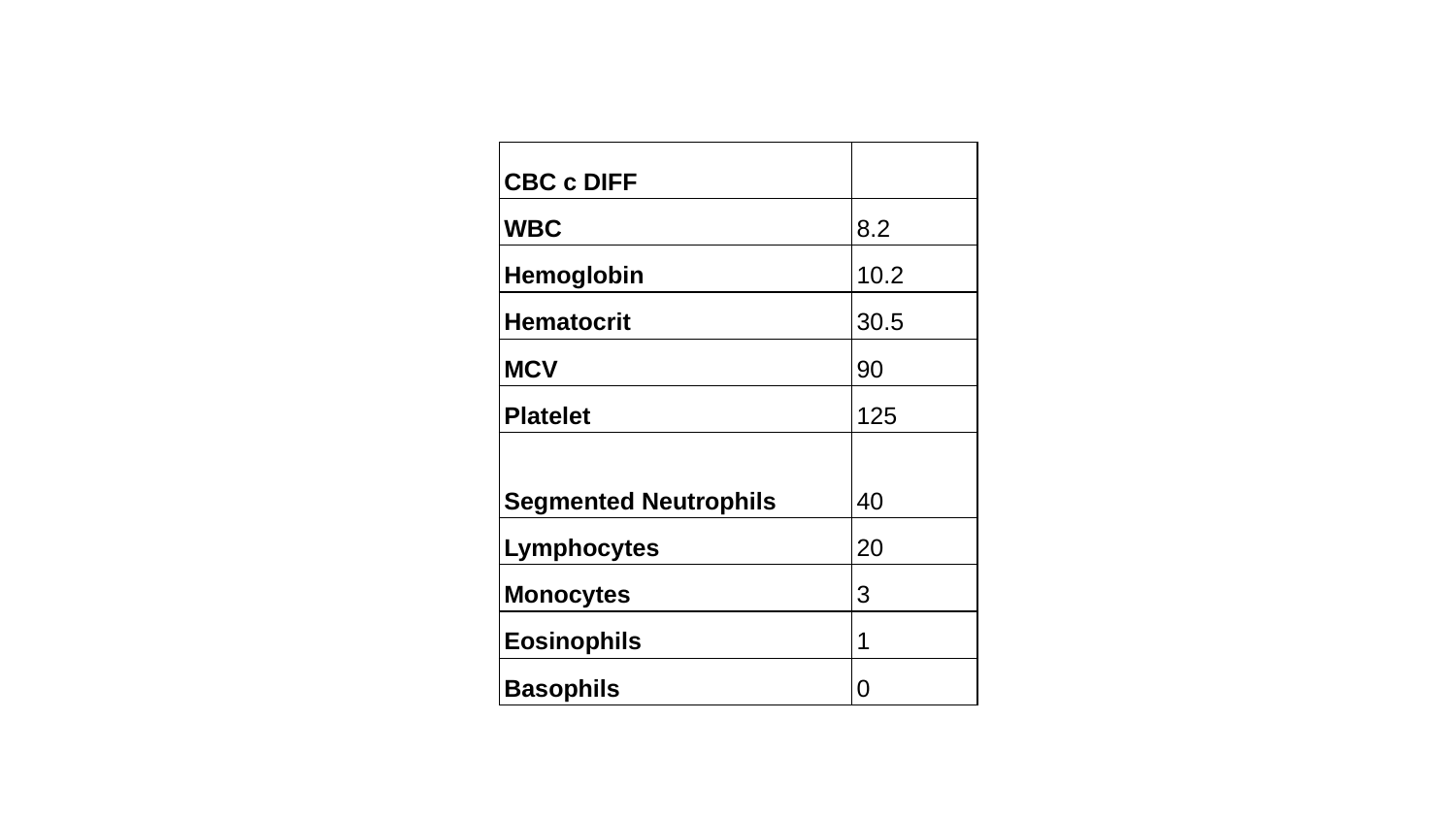

#
| CBC c DIFF | |
| --- | --- |
| WBC | 8.2 |
| Hemoglobin | 10.2 |
| Hematocrit | 30.5 |
| MCV | 90 |
| Platelet | 125 |
| Segmented Neutrophils | 40 |
| Lymphocytes | 20 |
| Monocytes | 3 |
| Eosinophils | 1 |
| Basophils | 0 |

## Slide 4
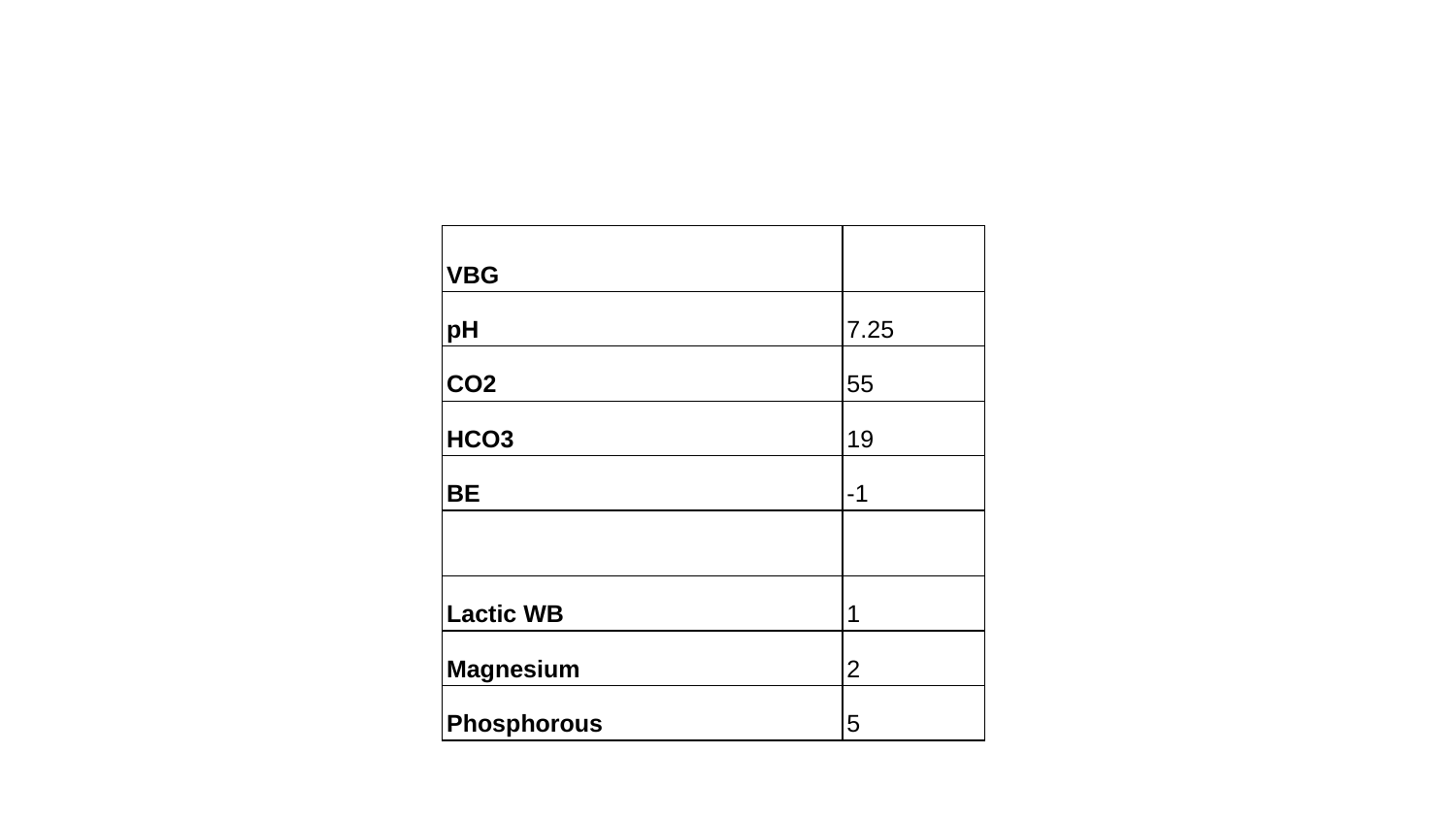

#
| VBG | |
| --- | --- |
| pH | 7.25 |
| CO2 | 55 |
| HCO3 | 19 |
| BE | -1 |
| | |
| Lactic WB | 1 |
| Magnesium | 2 |
| Phosphorous | 5 |

## Slide 5
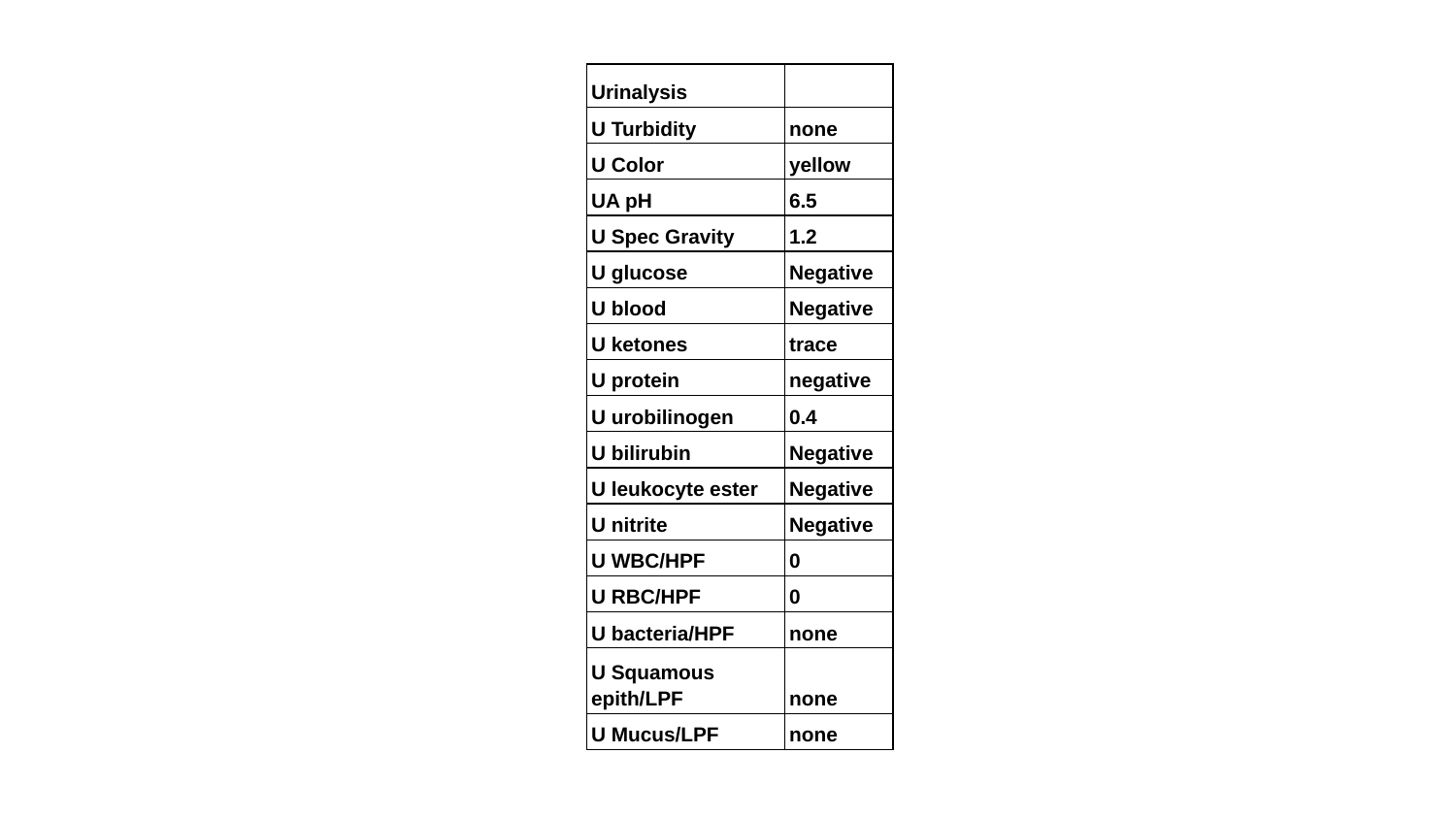

| Urinalysis | |
| --- | --- |
| U Turbidity | none |
| U Color | yellow |
| UA pH | 6.5 |
| U Spec Gravity | 1.2 |
| U glucose | Negative |
| U blood | Negative |
| U ketones | trace |
| U protein | negative |
| U urobilinogen | 0.4 |
| U bilirubin | Negative |
| U leukocyte ester | Negative |
| U nitrite | Negative |
| U WBC/HPF | 0 |
| U RBC/HPF | 0 |
| U bacteria/HPF | none |
| U Squamous epith/LPF | none |
| U Mucus/LPF | none |
#

## Slide 6
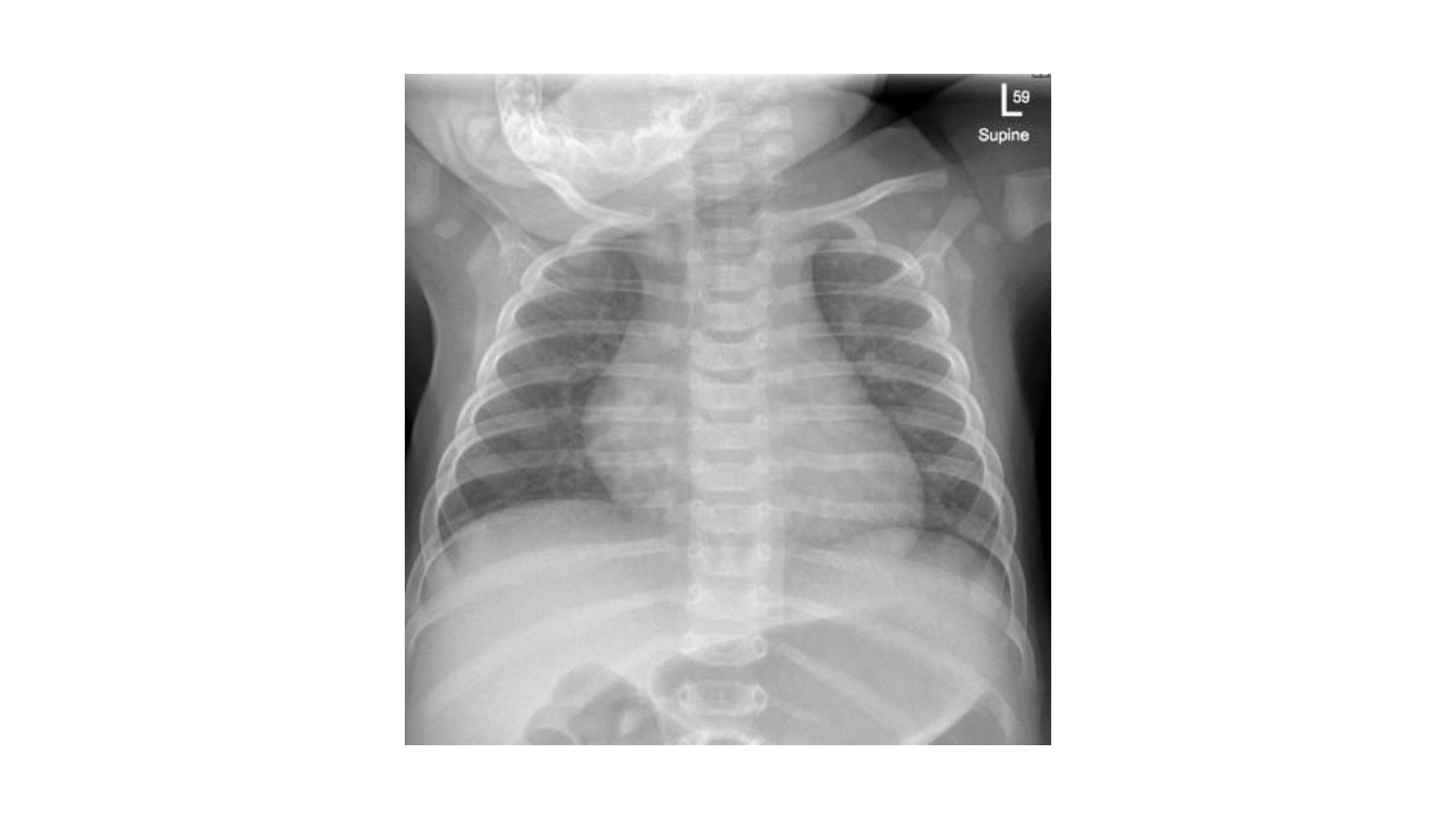

#

## Slide 7
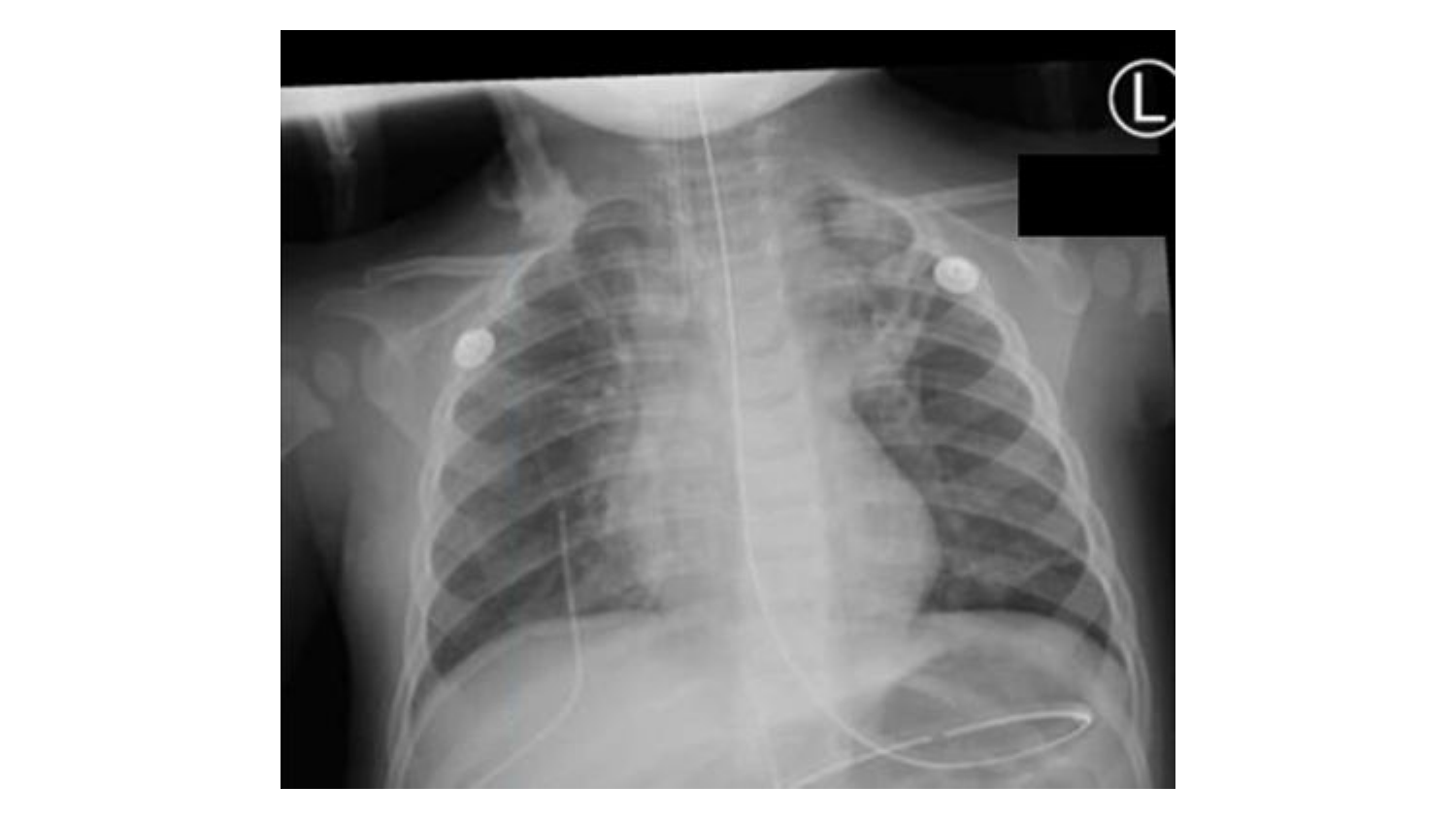

#

## Slide 8
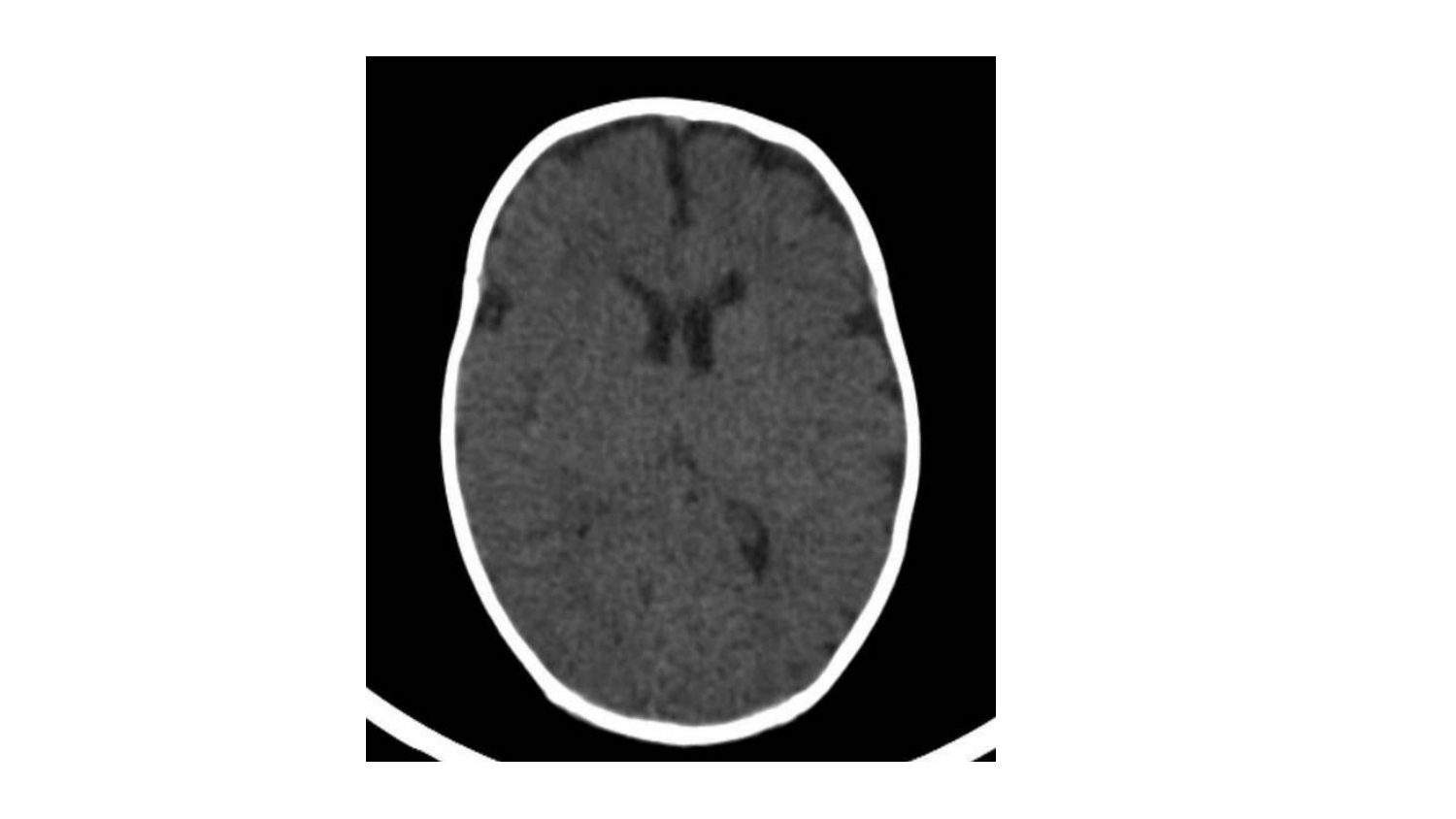

#

## Slide 9
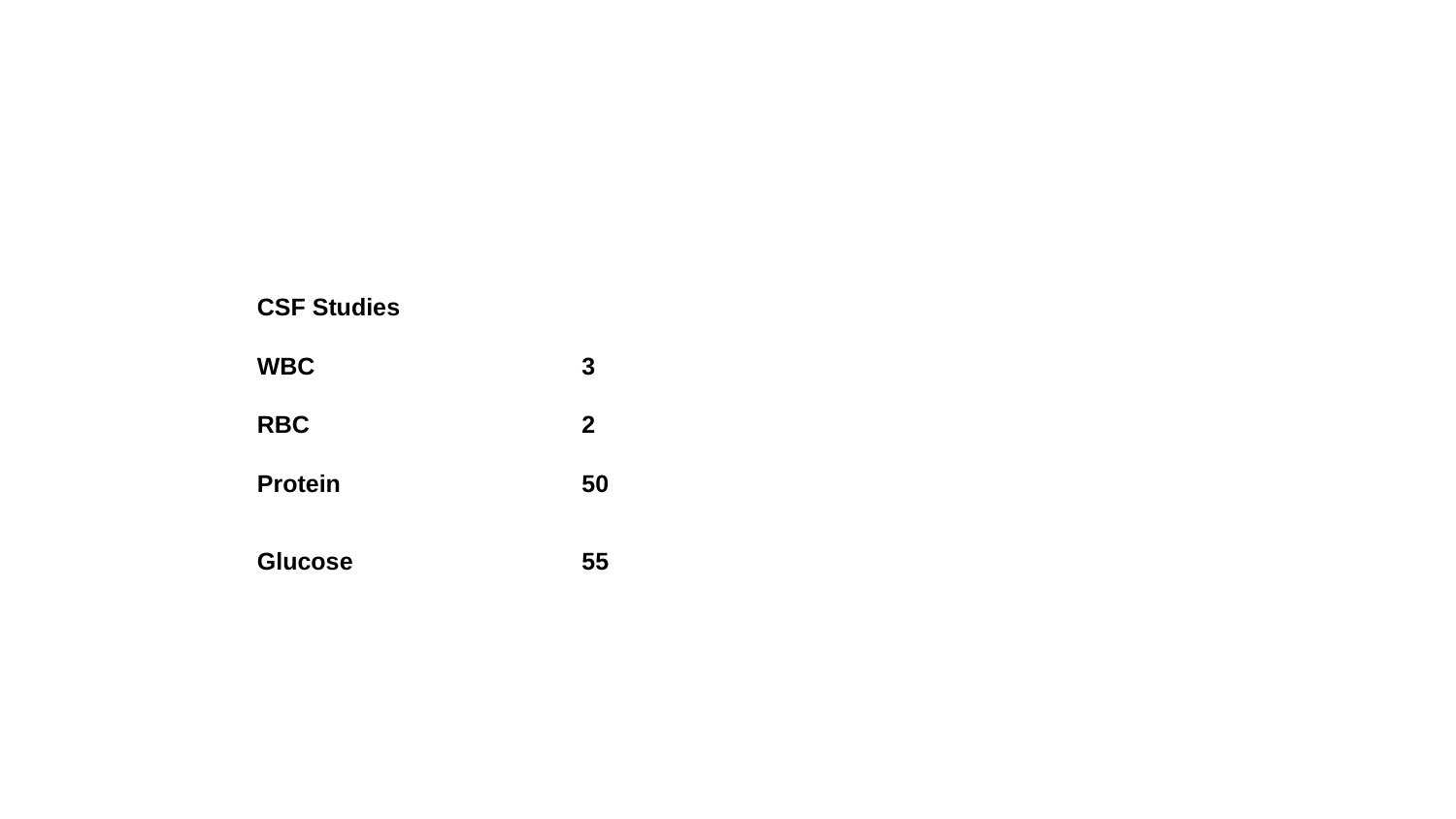

#
| CSF Studies | |
| --- | --- |
| WBC | 3 |
| RBC | 2 |
| Protein | 50 |
| Glucose | 55 |
